# Knot or not? Identifying unknotted proteins in knotted families with sequence‐based Machine Learning model

**DOI:** 10.1002/pro.4998

**Published:** 2024-06-18

**Authors:** Maciej Sikora, Eva Klimentova, Dawid Uchal, Denisa Sramkova, Agata P. Perlinska, Mai Lan Nguyen, Marta Korpacz, Roksana Malinowska, Szymon Nowakowski, Pawel Rubach, Petr Simecek, Joanna I. Sulkowska

**Affiliations:** ^1^ Centre of New Technologies, University of Warsaw Warsaw Poland; ^2^ Faculty of Mathematics, Informatics and Mechanics, University of Warsaw Warsaw Poland; ^3^ Central European Institute of Technology, Masaryk University Brno Czech Republic; ^4^ National Centre for Biomolecular Research, Faculty of Science, Masaryk University Brno Czech Republic; ^5^ Faculty of Physics, University of Warsaw Warsaw Poland; ^6^ Warsaw School of Economics Warsaw Poland

**Keywords:** AlphaFold, deep learning, knotted proteins, protein topology, SPOUT family proteins

## Abstract

Knotted proteins, although scarce, are crucial structural components of certain protein families, and their roles continue to be a topic of intense research. Capitalizing on the vast collection of protein structure predictions offered by AlphaFold (AF), this study computationally examines the entire UniProt database to create a robust dataset of knotted and unknotted proteins. Utilizing this dataset, we develop a machine learning (ML) model capable of accurately predicting the presence of knots in protein structures solely from their amino acid sequences. We tested the model's capabilities on 100 proteins whose structures had not yet been predicted by AF and found agreement with our local prediction in 92% cases. From the point of view of structural biology, we found that all potentially knotted proteins predicted by AF can be classified only into 17 families. This allows us to discover the presence of unknotted proteins in families with a highly conserved knot. We found only three new protein families: UCH, DUF4253, and DUF2254, that contain both knotted and unknotted proteins, and demonstrate that deletions within the knot core could potentially account for the observed unknotted (trivial) topology. Finally, we have shown that in the majority of knotted families (11 out of 15), the knotted topology is strictly conserved in functional proteins with very low sequence similarity. We have conclusively demonstrated that proteins AF predicts as unknotted are structurally accurate in their unknotted configurations. However, these proteins often represent nonfunctional fragments, lacking significant portions of the knot core (amino acid sequence).

## 1 INTRODUCTION

**FIGURE 1:**
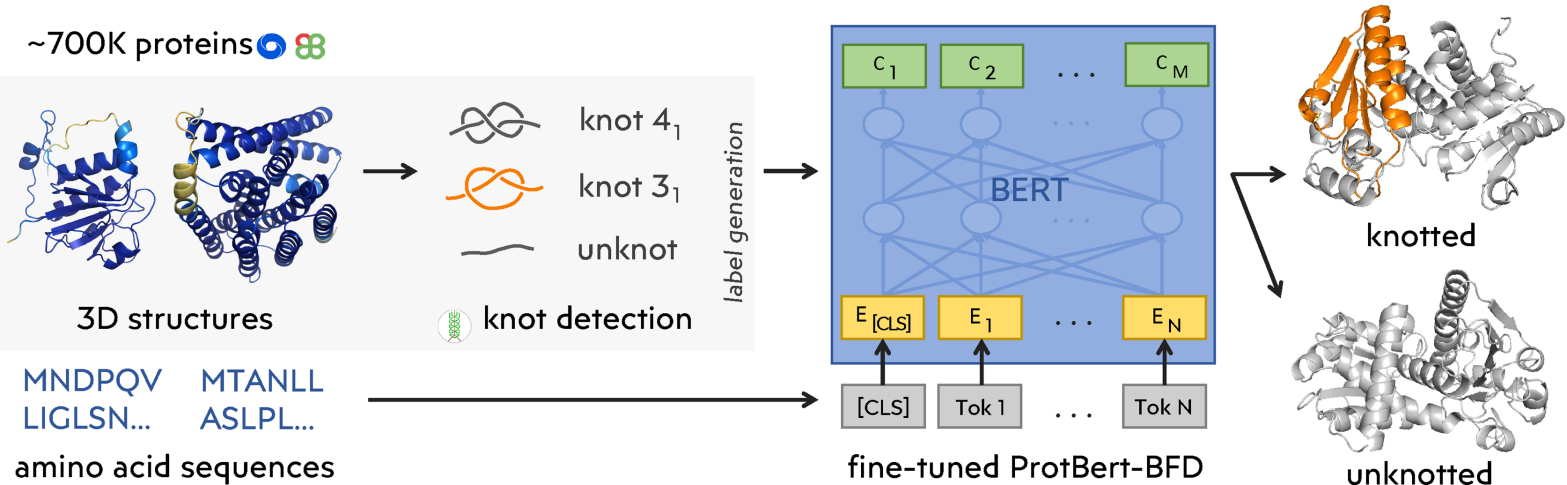
Scheme of the machine learning workflow. The entire approach consists of three main steps: 1. Obtaining data from PDB (with support of Knotprot) and AlphaFold (with support of AlphaKnot) predictions for UniProt; 2. Identifying nontrivial topology in the protein backbone using Topoly; 3. Training a machine learning model to predict nontrivial topology from the protein sequence.

The central paradigm of structural biology is the relationship between protein sequence, the 3D structure it encodes, and its biological function. The 3D structure is composed of structural motifs found consistently in unrelated and often sequentially distinct proteins. One motif type whose role is still unknown is a knotted motif, even though it was discovered three decades ago (Mansfield, 1994; Richardson, 1977; Takusagawa & Kamitori, 1996). The knots are formed by the protein backbone, and the simplest one, with three crossings (denoted as $3_{1}$), is shown in Figure 1.

Knots are a common part of our everyday life. In a macromolecular world, they easily form on polymers; however, the analysis of Protein Data Bank (PDB) shows, that knots are rare in the case of proteins (Dabrowski‐Tumanski et al., 2018; Virnau et al., 2006). Nevertheless, entangled proteins are found in species from all superkingdoms (Bacteria, Eukaryota, and Archaea alike; Hsu, 2023; Niemyska et al., 2022; Sulkowska, 2020), and conduct numerous functions in various parts of the cell (Niemyska et al., 2022). Knots are an integral structural feature of certain protein families (Christian et al., 2016; Tkaczuk et al., 2007) where they can form an active site. Moreover, they are exceptionally conserved, even in families with high sequence diversity (Potestio et al., 2010; Sułkowska et al., 2012; Zayats et al., 2021). Knotted proteins have already been investigated from different perspectives: folding (a Beccara et al., 2013; Baiesi et al., 2019; Bustamante et al., 2017; Faísca, 2015; Flapan et al., 2019; Jackson et al., 2017; King et al., 2010; Lim & Jackson, 2015; Neelamraju et al., 2022; Silva et al., 2022; Škrbić et al., 2012; Sulkowska, Sulkowski, & Onuchic, 2009; Wallin et al., 2007; Wang et al., 2015), degradation (Fonseka et al., 2021; Rivera et al., 2020; Wang & Li, 2020), thermal or mechanical stability (Rivera et al., 2020; Sulkowska, Sulkowski, Szymczak, & Cieplak, 2009; Wojciechowski et al., 2016), and potential biological function of the knot (Christian et al., 2016; Dabrowski‐Tumanski et al., 2016; Strassler et al., 2022; Sulkowska, Sulkowski, Szymczak, & Cieplak, 2009). However, despite broad efforts, the origin, the pattern of amino acids responsible for the entanglement, or the role of the knot is still unknown (Jackson et al., 2017). One of the main reasons for this is the amount of data being too limited for the confident identification of correlations between potential patterns and knotting behavior.

With the update of the AlphaFold (AF) database in 2022 (Jumper et al., 2021; Varadi et al., 2022), the structure predictions for all proteins from the UniProt database (nearly 200 million entries) became available. Therefore, knot detection and identification entered the Big Data age. The predictive quality of AF methods is already widely recognized as many models passed experimental confirmation (da Silva et al., 2023; Ferrario et al., 2022). Although knotted proteins account for only 1% of the structures deposited in the PDB, recent studies suggest, that the AF performs very well here too. AF has demonstrated accurate prediction of knots in known knotted families including homologs with very low sequence similarity (Niemyska et al., 2022), and a new type of knots (Brems et al., 2022; Doyle et al., 2023; Hou et al., 2023; Perlinska et al., 2023), in particular a composite knot (3_1_#3_1_; Brems et al., 2022; Nguyen et al., 2023; Niemyska et al., 2022), and a 7_1_ knot (Brems et al., 2022; Niemyska et al., 2022), whose existence has been experimentally confirmed afterwards (da Silva et al., 2023; Hsu et al., 2024).

Herein, we conducted a comprehensive review of all 200 million entries to establish the number of proteins with knotted AF models. We identified new potentially knotted families and focused especially on those in which different types of topology are predicted. Overall, there are ~700,000 knotted proteins (with high‐quality prediction—average full‐chain predicted Local Distance Difference Test [pLDDT] value above 70). We classified these proteins into 15 groups (superfamilies), including 10, whose members have experimentally determined knotted structures. However, even though it is expected that the knot is strictly conserved in the homologous proteins, we found that each group contains both knotted and unknotted members. Up until now, two types of topology have been observed only in the case of OTC/ATC family. These data appear surprising at first, but it enables us to use a Big Data approach to analyze knotted and unknotted proteins for the first time and allows us to ask some fundamental questions: (i) Is it possible to distinguish between knotted and unknotted proteins based solely on their amino acid sequences? (ii) In which protein families is the knot fully conserved? (iii) Does the sequence of a knotted protein contain a pattern responsible for its knotting?

Motivated by the significant accomplishments of large protein language models in other fields, herein we utilize this dataset to develop a ML model capable of accurately predicting the presence of a knot in a protein from its sequence. Next, we test the capabilities of the model by applying it to 100 proteins without structures predicted yet by AF and manually verify the results.

Next, to understand the performance of our ML model, we focus on individual families with and without experimentally verified knotted 3D structures. We ask whether the existence of mixed topology within one family is accurate or if it is a matter of the quality of AF prediction. We investigate all 15 groups from our dataset, including: SPOUT—the biggest knotted family which possesses $3_{1}$ and $3_{1}\#3_{1}$ knot structures (da Silva et al., 2023); sodium/calcium exchanger integral membrane proteins—a family of transmembrane proteins and deep $3_{1}$ knot; UCH—a big family with $5_{2}$ knot (Andersson et al., 2009); and ATC/OTC family with both knotted (deep $3_{1}$ knot) and unknotted topologies (Virnau et al., 2006) confirmed by x‐ray structures. Among other things, this analysis reveals new families composed of knotted and unknotted homological proteins. On the other hand, we show that a knot is strictly conserved in all other families.

Finally, we apply the ML model to seek answers to one fundamental question, undertaken repeatedly in the past: is there an amino acid sequence that is responsible for the knotting? The answer may uncover the physical reasons behind the knotting or evolution of the knotted proteins and pave the way to the design of artificial knotted proteins. We specifically focus on the SPOUT superfamily, which holds significant potential for industrial and pharmaceutical applications, such as the design of new antimicrobial drugs (Strassler et al., 2022).

## 2 RESULTS AND DISCUSSION

### 2.1 Identification of all potentially knotted proteins in the UniProt database based on AF predictions

In order to investigate what percentage of proteins predicted by AF is potentially knotted, we determined the topology of 178,015,209 structures with an average full‐chain pLDDT >70 out of all 214,683,829 structures predicted by AF (83%). The topology was detected using the Topoly package (Dabrowski‐Tumanski et al., 2020) with the HOMFLY‐PT polynomial and with probabilistic closer approach (Freyd et al., 1985; Przytycki & Traczyk, 2016; see Section 5.1.1 for details).

We found that knotted proteins occur in <0.35% (around 700,000) of all sequences deposited in the UniProt database. Using the cluster database at high identity with tolerance (CD‐HIT) clustering method, biological and functional annotations, and manual verification, we found that the majority of sequences can be classified into 15 superfamilies, given in Table 1. Those include families with well‐known knotted members such as SPOUT, AdoMet synthase, Carbonic anhydrase, or UCH families (9 families listed in Table 1; Jarmolinska et al., 2019; Niemyska et al., 2022); new families with $5_{2}$ knot‐like OPT (oligopeptide transporter; with already experimentally confirmed structures); families identified already by others with new types of knots, for example, $6_{3}$ but without experimental verification (Brems et al., 2022; Perlinska et al., 2023; Sikora et al., 2023); and yet new (not spotted before) families such as DUF2254 (IPR018723) or DUF4253 (IPR025349). We also found other knotted families (not listed in Table 1), which include members with more than three types of knots in homologous sequences (e.g., $0_{1}$, $3_{1}$, $5_{1}$, and $5_{2}$), they are deposited in the AlphaKnot Database (Niemyska et al., 2022) which collects all nontrivial proteins predicted by the AF Database (Perlinska et al., 2023), very complex knots with nine crossings (see AlphaKnot Database) and with <500 members. Details about classification into families can be found in Section 5.

**TABLE 1:** Protein families and domains of the dataset.

Superfamily	Family/domain name	Family/domain ID	AlphaFold		PDB		
			No. knotted models	No. unknotted models	No. knotted proteins	Example structure: Knotted	Example structure: Unknotted
SPOUT	tRNA (guanine‐N1‐)‐methyltransferase, N‐terminal	IPR029026	260,689 (3_1_)	8931	84 (3_1_)	1uak (3_1_)	‐
	tRNA methyltransferase TRM10‐type domain superfamily	IPR038459	4468 (3_1_)	1330	6 (3_1_)	4fmw (3_1_)	‐
ITIH	von Willebrand factor, type A	IPR002035	14,299 (6_3_; 3_1_)	209,414	0 (6_3_; 3_1_)	‐	1jey
	VIT domain	IPR013694	8118 (6_3_; 3_1_)	6717	0 (6_3_; 3_1_)	‐	6fpy
ATC/OTCase	Aspartate/ornithine carbamoyltransferase	IPR036901; IPR006131	6089 (3_1_)	81,789	5 (3_1_)	2 fg7 (3_1_)	1d09
‐	Sodium/calcium exchanger (membrane)	IPR004837	48,685 (3_1_)	20,450	4 (3_1_)	3v5s (3_1_)	‐
AdoMet synthase	S‐adenosylmethionine synthetase	IPR022628; IPR002133; IPR022636	42,982 (3_1_)	5846	18 (3_1_)	1fug (3_1_)	‐
	S‐adenosylmethionine synthetase, archaea	IPR027790; IPR042544; IPR002795	694 (3_1_)	226	4 (3_1_)	4hpv (3_1_)	‐
Carbonic anhydrase	Alpha carbonic anhydrase	IPR036398	19,202 (3_1_)	8487	33 (3_1_)	6y74 (3_1_)	‐
	Delta carbonic anhydrase	IPR018883	641 (3_1_)	111	‐	‐	‐
‐	Oligopeptide transporter	IPR004813	21,909 (5_2_)	7067	1 (5_2_)	7wsr (5_2_)	‐
‐	Integrin alpha	IPR032695; IPR013649	3799 (3_1_)	9053	1* (3_1_)	4cak* (3_1_)	7usl
TDD	tRNA‐uridine acp‐transferase	IPR005636	11,288 (3_1_)	776	‐	‐	‐
	16S/18S rRNA acp‐transferase Tsr3	IPR007209; IPR022968; IPR007177	3292 (3_1_)	747	2 (3_1_)	5ap8 (3_1_)	‐
‐	Calcium‐activated potassium channel BK, alpha subunit	IPR003929	4888 (3_1_)	3454	4 (3_1_)	3mt5 (3_1_)	‐
UCH	Ubiquitin carboxyl‐terminal hydrolase	IPR001578; IPR036959	6770 (5_2_)	3712	8 (5_2_)	1uch (5_2_)	‐
‐	DUF2254 (membrane)	IPR018723	1944 (3_1_)	5471	‐	‐	‐
‐	Lantibiotic dehydratase	IPR006827	2395 (3_1_)	2665	2 (3_1_)	5ehk (3_1_)	‐
‐	Ribosomal protein L37/S30	IPR010793	1242 (3_1_)	651	4 (3_1_)	3j7y (3_1_)	‐
‐	DUF4253	IPR025349	1546 (3_1_)	494	‐	‐	‐

*Note*: In parentheses are the types of knots found in PDB structures and AlphaFold models. In the case of models, the dominant knot types are given. In the case of knotted proteins with PDB structures, the number of proteins with topology specified in parentheses is provided. Asterisks indicate that the topology is uncertain due to gaps in key regions of the structures.

An intriguing observation is that each family contained both knotted and unknotted predictions, which was only observed in the case of ATCase/OTCase family (and confirmed experimentally). In the case of ATCase/OTCase family, AF predicts only 11% of knotted proteins of a set of 87,878 sequences. The most unexpected are cases of families where the knot was expected to be strictly conserved, for example, SPOUT family. Later on, we will show that in some cases we can explain these surprising results.

The data from Table 1 present a great deal of information that can be analyzed in a variety of ways, however, because it includes families with knotted and unknotted members (with high pLDDT) it presents an ideal dataset for the application of ML.

### 2.2 Dataset creation: For ML investigation

To investigate ML's ability to detect a knotted protein based on amino acid sequence, we focus on the biggest families (so families with $4_{1}$ and $6_{1}$ knot types have been omitted here). Thus our set includes 15 groups, where SPOUT, ITIH, AdoMet, Carbonic anhydrase, and TDD are represented by two InterPro IDs (in total 20 families). The data in Table 1, however, might include groups of proteins with potentially high sequential similarity. Thus to reduce the bias we filtered out the redundant data by applying sequential clustering. After processing we used 157,644 sequences for training and 39,412 sequences for testing (Table 2; further details are outlined in Section 5).

**TABLE 2:** Evaluation of the fine‐tuned ProtBert‐BFD model on the test set.

Protein superfamily	Dataset size	Unknotted part size	Accuracy (%)	TPR (%)	TNR (%)
All	39,412	19,718	98.5	98.7	98.3
SPOUT	7371	550	98.9	99.5	90.9
ITIH	14,263	12,555	98.7	94.2	99.3
ATCase/OTCase	3799	3352	100.0	99.8	100.0
Sodium/calcium exchanger	5256	726	99.0	99.7	95.3
AdoMet synthase	1794	240	99.0	99.3	97.1
Carbonic anhydrase	1531	539	95.9	97.4	93.1
Oligopeptide transporter	2510	456	98.7	99.5	94.7
Integrin alpha	332	224	83.1	66.7	91.1
TDD	612	24	99.0	99.7	83.3
Calcium‐activated potassium channel BK, alpha subunit	127	87	89.0	97.5	85.1
UCH	477	125	90.6	96.0	75.2
DUF2254 membrane	593	376	99.8	99.5	100.0
Lantibiotic dehydratase	392	286	96.4	95.3	96.9
Ribosomal protein L37/S30	147	41	85.7	100.0	48.8
DUF4253	123	53	86.2	90.0	81.1

*Note*: Accuracy, true positive rate (TPR), and true negative rate (TNR) of the model evaluated for the whole test set and per protein family.

### 2.3 ML classifies proteins as knotted or unknotted

Next, we ask if there is a ML model capable of solving the binary classification of proteins into knotted and unknotted classes based solely on their amino acid sequences.

This inquiry was based on the preprocessed dataset, encompassing 157,644 training and 39,412 testing entries (Table 2). The dataset consists of knotted and unknotted amino acid sequences from 20 different protein families with various knot types (with the prevalence of $3_{1}$ knot). Combining the dataset from individual protein families implies that the dataset is diverse. Sequences within one family are typically similar to each other, but diverse from proteins from other families and each family usually has a different biological function. The sequences also show differences across families in features such as average sequence length or average knot core size. During the evaluation, we thus approach the dataset from two perspectives: the first is a Big Data approach, where we treat the data as a whole and test the performance of the model on a big scale, and the second is a more detailed analysis and interpretation of results for individual selected families.

We decided to train three distinct models for the task of knot detection, where each model incorporated a progressively less complex architecture and strategy. This approach allowed us to compare the performance of the models and select the most optimal one. Furthermore, it enabled us to investigate whether solving the problem necessitates the use of a large, complex model or if a simpler approach suffices. The first model was a fine‐tuned ProtBert‐BFD (Elnaggar et al., 2022) network, the second was a convolutional neural network using ProtBert‐BFD embeddings as input, and the third was a simple convolutional neural network that operated on sequences with one‐hot encoded amino acids (further details can be found in Section 5). Refer to Figure 1 for a summary of the dataset creation process and model training.

After applying ML to our testing dataset (Table 2), we found that it is possible to distinguish knotted from unknotted proteins based solely on their amino acid sequence. All models showed robust results on the test set (accuracy above 95%), with the first model, the fine‐tuned ProtBert‐BFD model, demonstrating the best overall accuracy of 98.5%. As our dataset consists of a collection of distinct protein families, where proteins from different families differ in various respects, assessment of models across individual families is necessary. After the evaluation, we chose the first fine‐tuned ProtBert‐BFD model for subsequent analysis as it displayed strong accuracies across all the different protein families (see Table 2). We attribute the superior performance of the final model to the usage of a pretrained protein language model. As we can see on its visualized t‐SNE embeddings in Figure S1, the model already contained some information about the protein knotting status even before fine‐tuning it for the knotting task.

In the following sections, we will first test the ML model on the selected set of 100 protein sequences not predicted yet by AF. Next, we focus on individual protein families and delve into a qualitative examination of the chosen model.

### 2.4 Verification of the model on sequences without AF prediction

To further verify the ability of the model to correctly predict whether the protein is knotted or unknotted, a new testing dataset was created. The best dataset for this testing is protein sequences not yet analyzed or predicted by AF.

In the UniProt Database, there are around 50 million proteins without a structure predicted by the AF model (around 20%). Among those, we determined that around 240,000 records may constitute part of the families analyzed in this work (Table 1). For each family, we selected five proteins, in total of 100 sequences (details in Section 5), and predicted their topology with our model. Given that the proteins do not have their structures available in databases, we predicted 3D structure with locally installed AF 2 (five models each, to be sure that the results are robust from the topological point of view) and calculated their topology with the Topoly package. Independently, we analyzed the topology of the closest sequential homologs (based on all proteins deposited in the AF database) for each of the proteins. Taking all these results into account, we determined for each protein whether it should possess a knot. Our model was correct in 92% of the cases (Tables 3 and S3).

**TABLE 3:** Evaluation of the dataset of proteins without structure prediction in AlphaFold database.

Protein ID	Our model (score)	AlphaFold model	Max. identity to train set (%)	Homolog	Prediction
IPR029026 (SPOUT)					
A0A8H3XI24	0.9976	3_1_ (82.97)	30	3_1_ (94.4)	Correct
A0A2E9CFS3	0.7200	3_1_ (73.48)	32.5	3_1_ (31.1)	Correct
A0A8T6ML81	0.3252	0_1_ (79.14)	33.3	3_1_ (32.2)	Incorrect
A0A8J5EEA0	0.0013	0_1_ (41.67)	32.7	3_1_ (27.7) *	Correct
A0A968VFV8	0.0003	0_1_ (81.12)	29.7	3_1_ (57.8) *	Correct
IPR025349 (DUF4253)					
A0A939XUL2	0.9473	3_1_ (68.5)	‐	3_1_ (25.5)	Correct
A0A956ZEP6	0.9927	0_1_ (58.73)	‐	0_1_ (25.5)	Incorrect
A0A9E1HYU6	0.9750	3_1_ (95.46)	82.2	3_1_ (93.2)	Correct
A0A8J8AKZ2	0.9946	0_1_ (53.07)	‐	0_1_ (22.1)	Incorrect
A0A956QTU2	0.0097	0_1_ (79.31)	‐	0_1_ (25.7)	Correct

*Note*: For each protein, we search for its closest homolog and report its topology and sequence identity (% in parenthesis). The asterisk indicates that the query protein does not contain the knotted core of its homolog and, thus is likely an unknotted fragment. For the AlphaFold models we generated for each query protein, we report their topology and global average pLDDT values (a value above 70 indicates the overall good quality of the model). The full table (100 proteins with detailed analysis) is available as Table S3.

Additionally, we verified that this high performance 92% of success, was not correlated with high identity between tested proteins and the train set. For 13 of the tested proteins, no similar proteins in the train set were found. For 26 tested proteins, we found train set proteins with an identity level below 50%. Results show, that there are numerous examples of correct predictions and low identity between the sets (<30%). In 90 cases (out of 100) the model and AF prediction give the same correct results. In two cases, the AF prediction seems to be incorrectly unknotted—our model predicts a knot and the homologs of these proteins are also knotted. These results show that our model is capable of predicting the topology of the proteins as well as the AF 2 does, for the families it was trained on. Although the data used to train the model contains unknotted proteins that are sometimes only fragments of knotted proteins. We will explain this apparent inconsistency in the next section. Conflicts between structural and sequential‐based topological assessments may also help detect artifacts in the AF Database. Given that the AF prediction can be time‐consuming, we believe that our model can be an alternative for fast check whether a protein is knotted or unknotted for a protein belonging to the selection of protein families used for training.

The remaining 8% of the proteins were mislabeled by our model. Most of these proteins are from different superfamilies which show that the predictive power of our model is not dependent on the selected group of proteins. Also, we did not observe a clear pattern behind the incorrect predictions. For example, in the case of the SPOUT superfamily our model and AF predicted a protein (UniProtKB ID: A0A8T6ML81) as unknotted, even though its knotted homolog exists (Table 3). Moreover, both methods predicted an almost identical protein (UniProtKB ID: A0A2E9CFS3; 98% identity) correctly as knotted. It is an interesting case for the future to analyze what factors impact the models' decisions. We observe a different situation in the DUF4253 group in which the model predicted two proteins as knotted even though they are probably unknotted as evidenced by the unknotted homologs and structure models we generated using AF. However, the verification methods yielded not highly decisive results (low sequence identity of the homologs and low pLDDT value of AF models), which shows the difficulty of these cases.

### 2.5 Analysis of unknotted proteins from all knotted families

Now, we focus on AF prediction—our selected dataset, which itself is very unexpected, and analyze it from a structural biology perspective. This analysis also aims to demonstrate the validity of the ML model. Note, that our dataset consists of protein families in which both knotted and unknotted proteins are predicted by AF with an average full‐chain pLDDT above 70. Moreover, 11 families include at least one protein with determined experimentally knotted 3D structures (see example in Table 1). This is quite unexpected since it was suggested that knot topology is strictly conserved in protein families (Sułkowska et al., 2012), and the only example of a mixed topology within a single family reported until now was the ATC/OTC family (Virnau et al., 2006). More specifically, within this family, several subfamilies with a conserved topology are indicated: the unknotted subfamilies include ATC, OTC, and PTC, while the knotted subfamilies involve AOTC, SOTC, and YTC (Shi et al., 2015). To ascertain whether the proteins with a given topology in our set belong to the appropriate subfamilies (i.e., whether the topology of the subfamilies is conserved), we clustered their sequences and aligned them to assign the closest homologs. This analysis showed that there are no topologically misclassified proteins from this family in our dataset—all unknotted proteins have the closest homologs from unknotted subfamilies and all knotted proteins from knotted subfamilies. Thus, our model can accurately differentiate between topologically different members of the ATC/OTC family. It is worth noting that there is an unknotted structure of a SPOUT protein deposited in the PDB database (PDB ID: 1oy5). This is a protein from a well‐studied TrmD family of $3_{1}$ knotted tRNA methyltransferases that is likely an error considering its knotted homologs and inability to function in an unknotted state (Christian et al., 2016; Jarmolinska et al., 2018).

**FIGURE 2:**
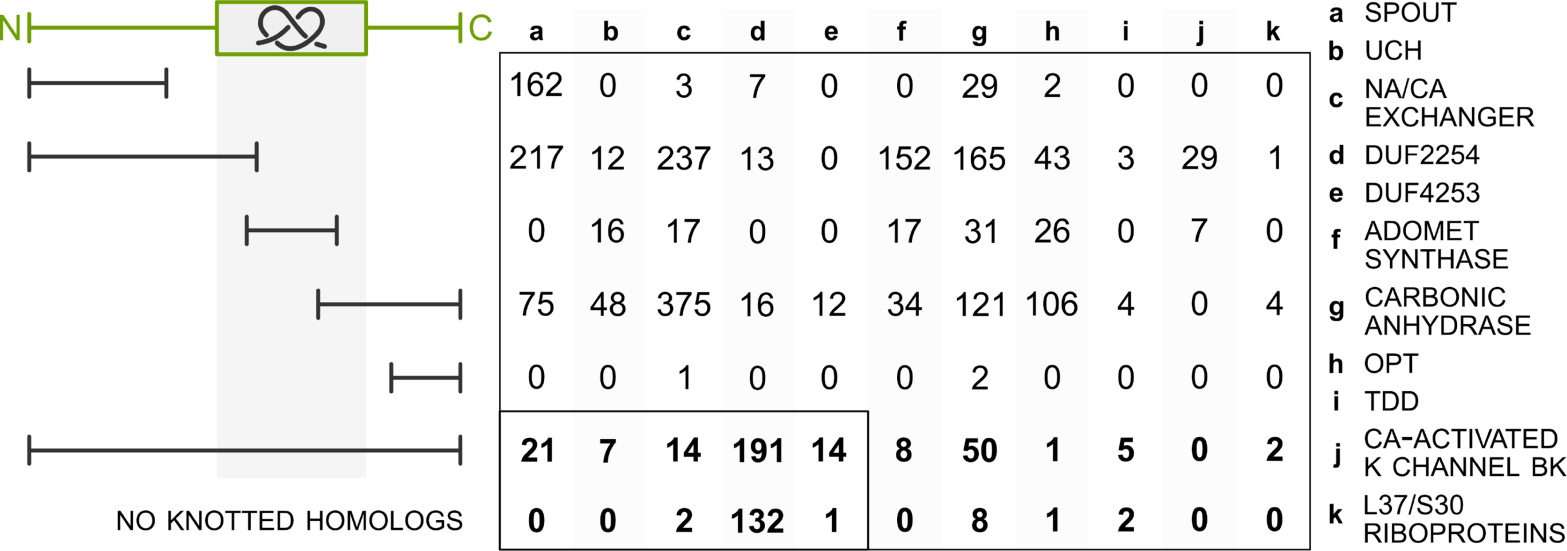
Unknotted proteins aligned with their knotted homologs. The schematic on the left represents the sequential alignment between knotted (green) and unknotted (black) proteins. The region with the knot is marked with a gray area. The unknotted proteins that start or end prematurely in comparison to their knotted counterpart are discarded from further analysis (see the section about SPOUT superfamily for details). We focus on cases in which the unknotted protein can be globally aligned with the knotted homolog (even with a deletion in the knot core) or does not have any knotted homologs (the numbers of these proteins are bolded).

Next, we carry out a very detailed analysis of unknotted structures (from the test set) of the remaining families to determine whether they are truly unknotted or simply a result of database inaccuracies, such as AF's incorrect structure prediction or fragmented sequences in UniProt. In particular, we seek unknotted proteins, which are full‐fledged members of their otherwise knotted family, meaning they possess a sequential (amino acid) equivalent of the knot, and they have the potential to be functional. For each family, we analyzed only the unknotted proteins from the test set (Table 2) which our model also predicted as unknotted. For all of these proteins, in the first step of the analysis, we search for the most similar protein that is knotted, a knotted homolog. Then, for each such pair, we determine if the unknotted protein has the potential to be truly unknotted by analyzing pairwise sequential alignment. If the unknotted protein has a sequence that is equivalent to the knot core of its homolog or does not have a knotted homolog at all, we analyze it in more detail to verify it (Figure 2). The detailed analysis was done on five families: SPOUT, UCH, sodium/calcium exchanger, DUF2254, and DUF4253. Note, that we did not analyze the superfamilies in which the majority of the protein models are unknotted, thus we excluded the ITIH (Sikora et al., 2023) and integrin superfamilies (Perlinska et al., 2023).

### 2.6 Families with potential of having both knotted and unknotted proteins

#### 2.6.1 DUF4253

All unknotted members of the DUF4253 family, except for two proteins, have a knotted homolog, with a low average sequence identity of 30.1%. While 44% of the unknotted proteins lack the C‐terminal part of the knot core, the rest possess amino acid sequences sufficient to create an entire knot core (Figure 2). It is worth mentioning that in the latter case, we observe a deletion of the knot core loop fragment. Unknotted proteins align relatively well to the knot tails of their nontrivial counterparts as well as the beginning and the ending of the knot core with high confidence scores (pLDDT), but then omit the formation of the twisted loop fragment (amino acid sequence), which is necessary part to form the nontrivial topology (visualization on Figure 4). Based on the structural alignment and the fact there are no crystal structures, we conclude that predicted unknotted DUF4253 proteins may have a true trivial knot. To further confirm that both knotted and unknotted proteins with this domain exist, we prepared a sequential clustering for all proteins from the DUF4253 family. In over 91% of the clusters at a 60% identity threshold, there were exclusively either knotted or trivial proteins within one cluster. Moreover, we verified that in the vast majority of the clusters, the root mean square deviation (RMSD) values of pairwise structural superpositions do not exceed 5 Å. This shows that these topologies are conserved both sequentially and structurally within this family, and therefore probably both exist.

#### 2.6.2 DUF2254

Among unknotted DUF2254 proteins, only 63% have a knotted homolog (with a low average sequence identity of 30.46%). In a few cases (25% of the unknotted proteins with a knotted homolog) lack of the entanglement was due to a missing C‐terminal or N‐terminal part of the knot core. However, in most examples, unknotted DUF2254 (191 proteins) members align with the entire knotted regions of their homologs (Figure 2). In this case, the structure predicted by AF has a slipknot topology (King et al., 2007; Sułkowska et al., 2012) formed by the C‐terminus (Figure 4). Similarly to the DUF4253 family, unknotted proteins have high pLDDT. For this set of proteins, we also conducted a sequential clustering. The results obtained for this family exceeded those achieved for the aforementioned DUF4253 family. At the identity threshold of 60%, all of the clusters had members with a uniform topology (there were either only knotted or unknotted proteins within one cluster). Based on the structural alignments, we also observe that there are only a few outliers with RMSD values higher than 5 Å when superimposed onto remaining family members. Therefore, also this case shows that both knotted and unknotted proteins can be a part of a single family. The summary of sequential clustering and statistics of structural analysis can be found in the Supplementary material S1.

#### 2.6.3 UCH

In the case of this superfamily, 17.4% of the proteins in our test set are unknotted even though strict conservation of the knotted regions has been shown, despite large sequence divergence between the proteins (Sułkowska et al., 2012). Our investigation revealed that all unknotted proteins have a close, knotted homolog (with an average sequence identity of 80.5%), and 92% of them are annotated as fragments. The unknotted proteins lack at least a portion of the knot core, making them unknotted (Figure 6). We analyzed seven proteins in detail and all of them have a deletion inside the knot core. Interestingly, due to the nature of the $5_{2}$ knot, cutting its N‐ or C‐terminus has different effects on the structure's topology. Elimination of the first N‐terminal crossing leads to an unknotted structure—the remaining chain can be easily straightened by untwisting. On the other hand, removing the first crossing from the C‐terminal side changes the knot type (to $3_{1}$) but does not unknot the structure. Figure 4c shows examples of models with different topologies we found of proteins from the UCH family. Six (out of seven) of the unknotted proteins we found miss the first N‐terminal crossing due to a deletion in the sequence. The seventh structure actually possesses a $3_{1}$ knot (UniProtKB ID: A0A0E0DGQ2; Figure 4c, middle). This inconsistency is probably due to it being a shallow knot that went under the radar of our probabilistic knot detection algorithm. This protein is sequentially identical to its homolog with a $5_{2}$ knot. The only difference is a deletion in the C‐terminal part that makes the shorter protein $3_{1}$ knotted. In fact, these proteins are isoforms that arise due to alternative splicing of the same gene. This is the first example, to our knowledge, of topologically distinct isoforms.

#### 2.6.4 Sodium/calcium exchanger transmembrane proteins

**FIGURE 3:**
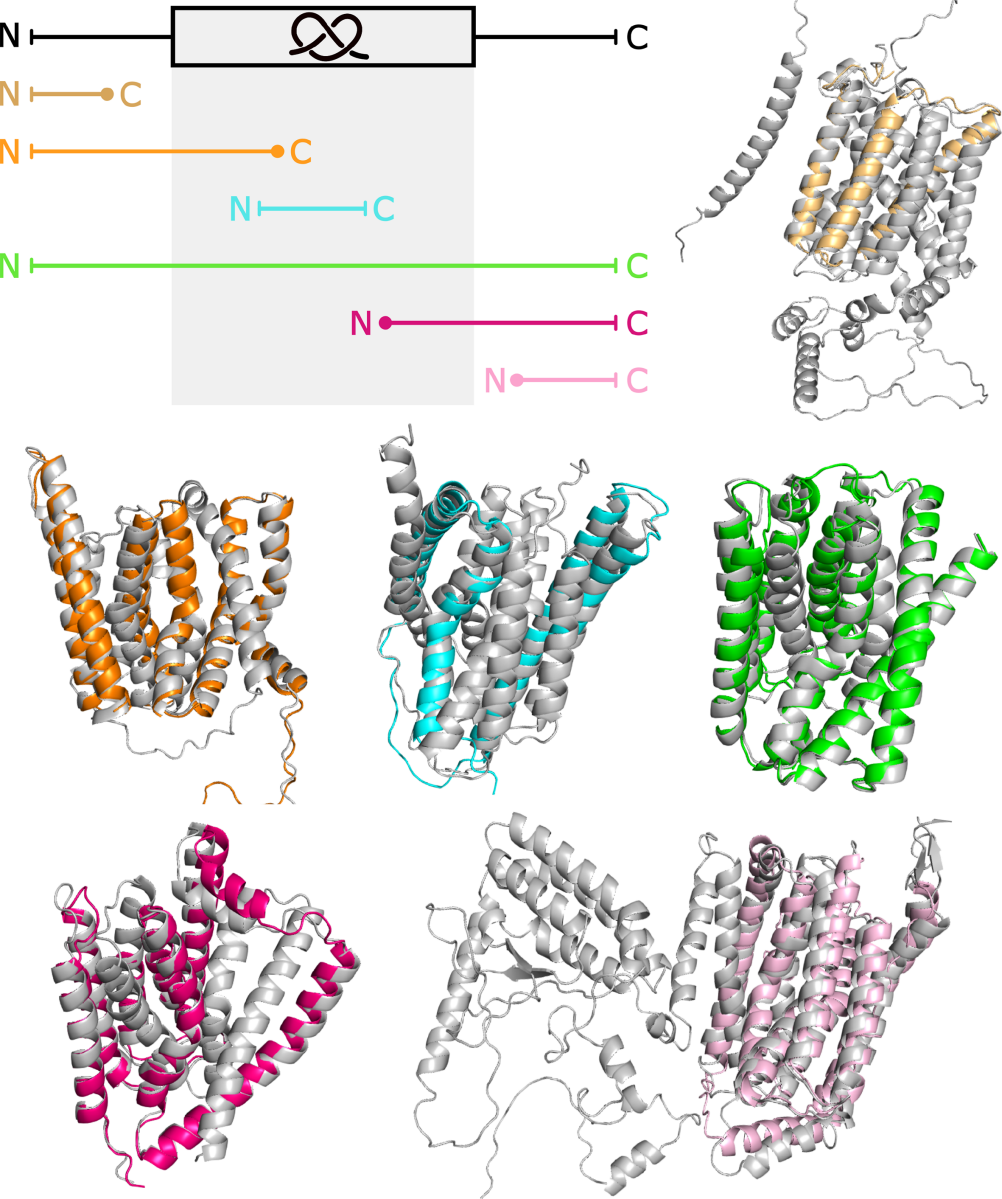
Unknotted membrane proteins from Sodium/calcium exchanger family superposed with their knotted homologs. Upper part: unknotted structure (light orange; UniProtKB ID: A0A5K0X7L3) without the entire knot core, unknotted structure (orange; UniProtKB ID: A0A397EWI1) structure lacking the C‐terminal part of the knot core, and unknotted structure (green; UniProtKB ID: A0A562DKV6) with low‐quality part of the knot core. Lower part: unknotted structure (cyan; UniProtKB ID: A0A453N937) without the N‐terminal and C‐terminal part of the knot core, unknotted structure (magenta; UniProtKB ID: A0A378FTV0) without the N‐terminal part of the knot core, and unknotted structure (light pink; UniProtKB ID: K1RUG8) without the entire knot core. All knotted homologs are shown with gray color (UniProtKB IDs: V4U0P6, A0A397A8U0, E6WVS2, A0A453N959, A0A0E1C8E8, and A0A0L8I7H8, respectively).

**FIGURE 4:**
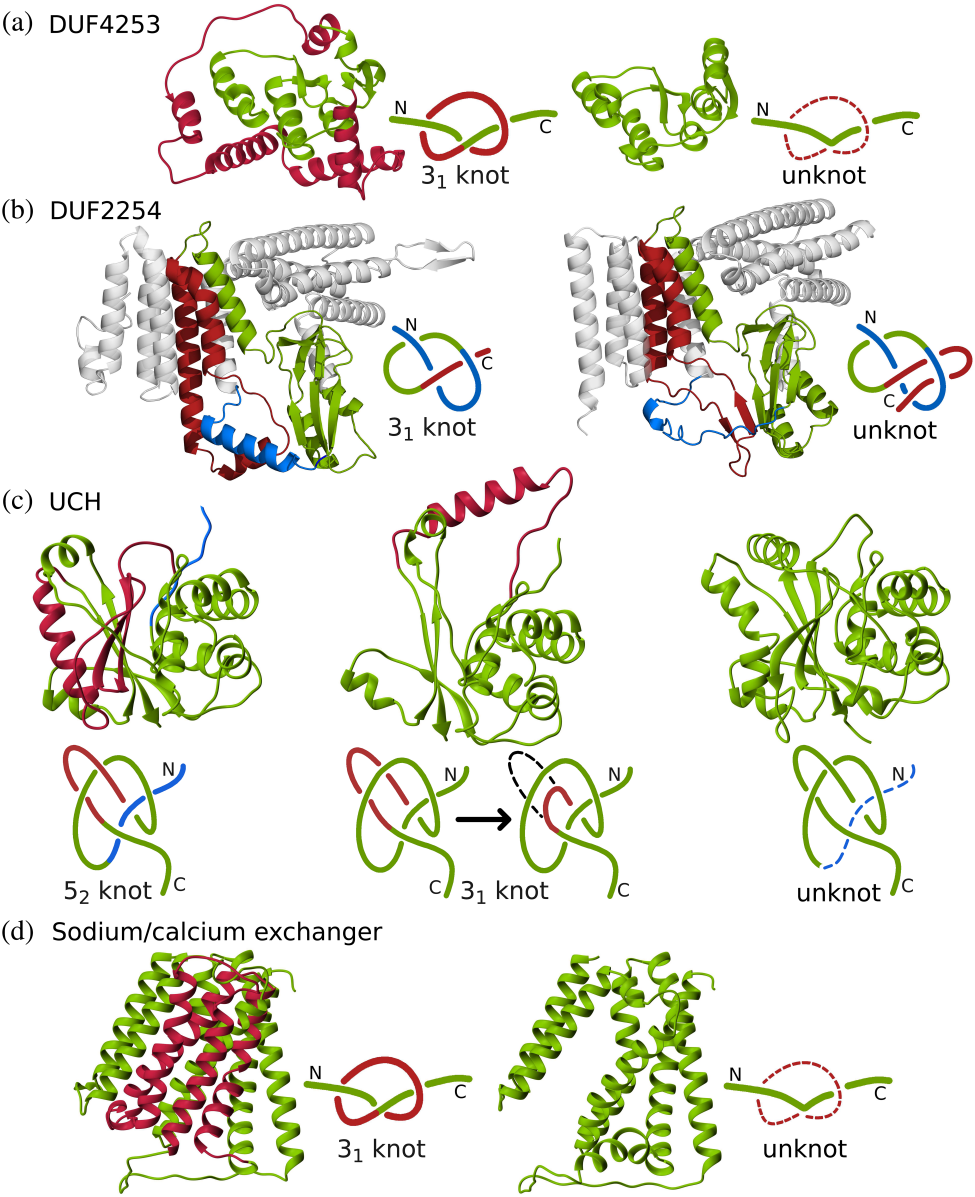
Overview of protein families with the potential of having both knotted and unknotted topologies. (a) Family DUF4253. A deletion (in red) aligning to the knot loop in the nontrivial homolog (UniProtKB ID: A0A1F0KRL5) creates an unknotted structure (B0C9T3). (b) DUF2254 family. The C‐terminus (in red) instead of passing through the loop (in blue) as in the knotted structure (A0A848U8E4), turns back creating a slipknot—an unknotted structure (D5C1U6). (c) UCH family. Compared with the standard structure with $5_{2}$ topology (A0A0E0DGQ1), either we observe a different arrangement of the loop committing one crossing (in red), which simplifies the topology to $3_{1}$ (A0A0E0DGQ2) or the fragment of N‐terminus is missing, which simplifies the topology to an unknot (A0A178ZI05). (d) Sodium/calcium exchanger transmembrane protein family. In the unknotted protein (A0A2G1VBT6) we observe the deletion of the fragment (in red) corresponding to the loop knot in the nontrivial homolog (A0A2S5ZE01).

This is the only family consisting of knotted membrane proteins (sodium/calcium exchanger integral membrane proteins; Jarmolinska et al., 2019), and also here a strict conservation of a knotted topology was suggested based on the available 3D structures (Zayats et al., 2021). All unknotted proteins have a close homolog that is knotted (with, on average, 79.5% sequence identity). Based on their pairwise alignment, most of the unknotted sequences either align with a fragment starting before the knot core and ending on a residue inside it, or with a fragment starting inside the knot core and ending after it (see Figure 3). Thus, due to the lack of a knot core sequence, these proteins cannot form the nontrivial topology. In this case, 56% of the unknotted proteins are annotated as fragments. However, we can also observe cases where alignment starts and ends outside of the knot core yet the protein is unknotted suggesting that also in this family both knotted and unknotted proteins can truly exist. This happens due to the unknotted structures missing a knot loop (example in Figure 4d).

### 2.7 Knotted families without truly unknotted proteins

#### 2.7.1 The SPOUT

The SPOUT superfamily is the largest knotted family in our dataset with known 3D structures. All of the proteins with experimentally determined 3D structure are knotted but AF predicts 7% of the entire group to be unknotted. We examined the correctness of part of these structures by analyzing their homologs: each protein was sequentially aligned with its closest knotted homolog to determine whether it contained a region corresponding to the knot core (Figure 2).

**FIGURE 5:**
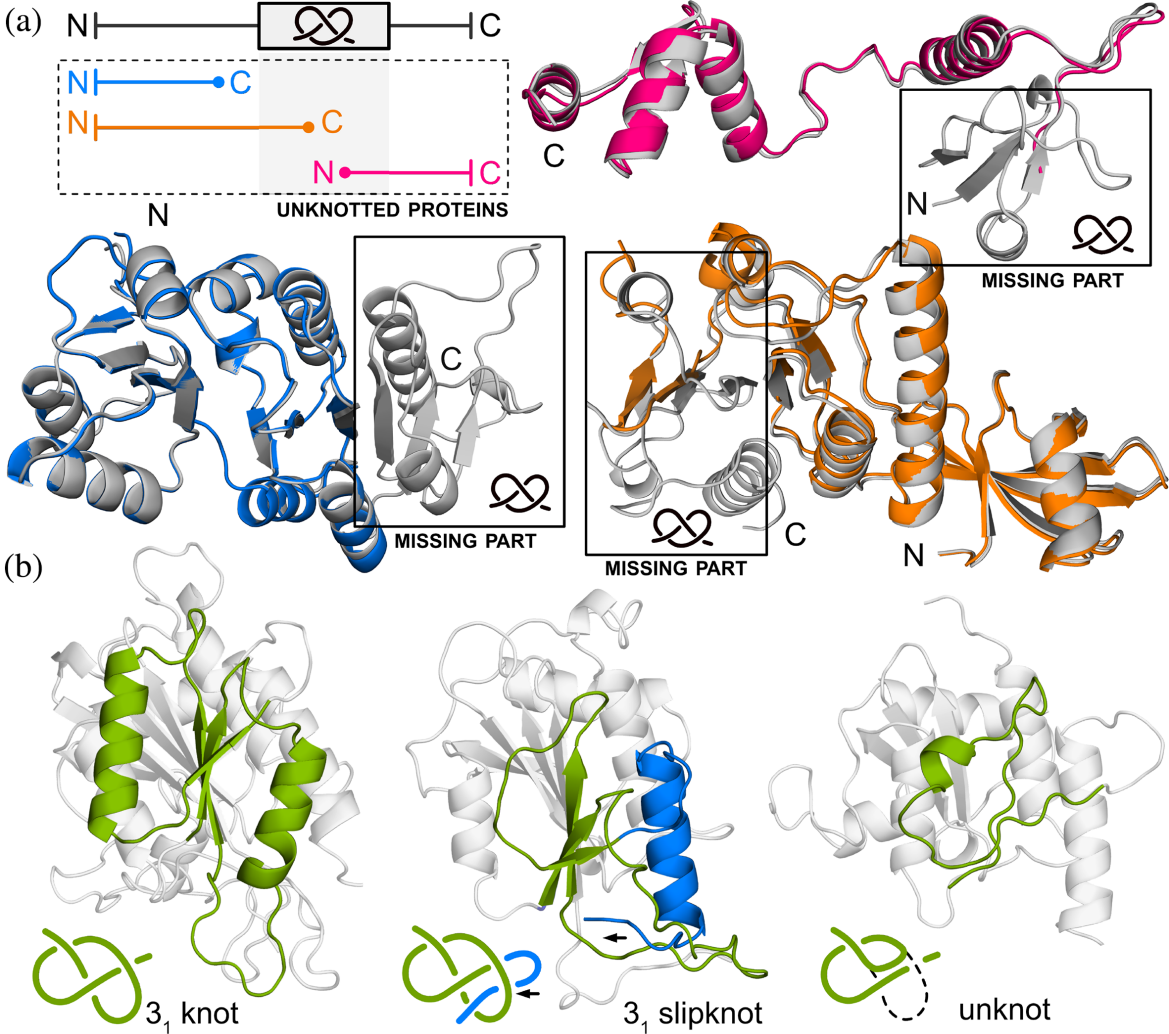
Unknotted SPOUT proteins (a) superposed with their knotted homologs. Upper part: unknotted structure (magenta; UniProtKB ID: A0A418H8R5) lacking the N‐terminal part of the knot core. Lower part: unknotted structure (blue; UniProtKB ID: A0A090WL68) without the entire knot core, and unknotted structure (orange; UniProtKB ID: A0A356CS99) without the C‐terminal part of the knot core. All knotted homologs are shown with gray color and their knotted regions are marked (UniProtKB IDs: A0A2X1NEC8, A0A090W861, and A0A3D0W667, respectively). Since all of these unknotted proteins lack at least a portion of the knot core, and the knot core is necessary for the SPOUT protein to function, we presume that these proteins are not truly unknotted. (b) Left: an example of a SPOUT protein with a typical $3_{1}$ knotted structure (UniProtKB ID: R1DT92). Middle: structure with a $3_{1}$ slipknot topology (UniProtKB ID: A0A424QRT9). The slipknot is formed by a C‐terminal end (colored blue) threaded back through the knot loop. The structure of the knot (colored green) remains typical for the SPOUT superfamily. Right: unknotted structure due to a deletion in the knot core (UniProtKB ID: R1FAN9). The presented structures are models from the AlphaFold database (4th version).

**FIGURE 6:**
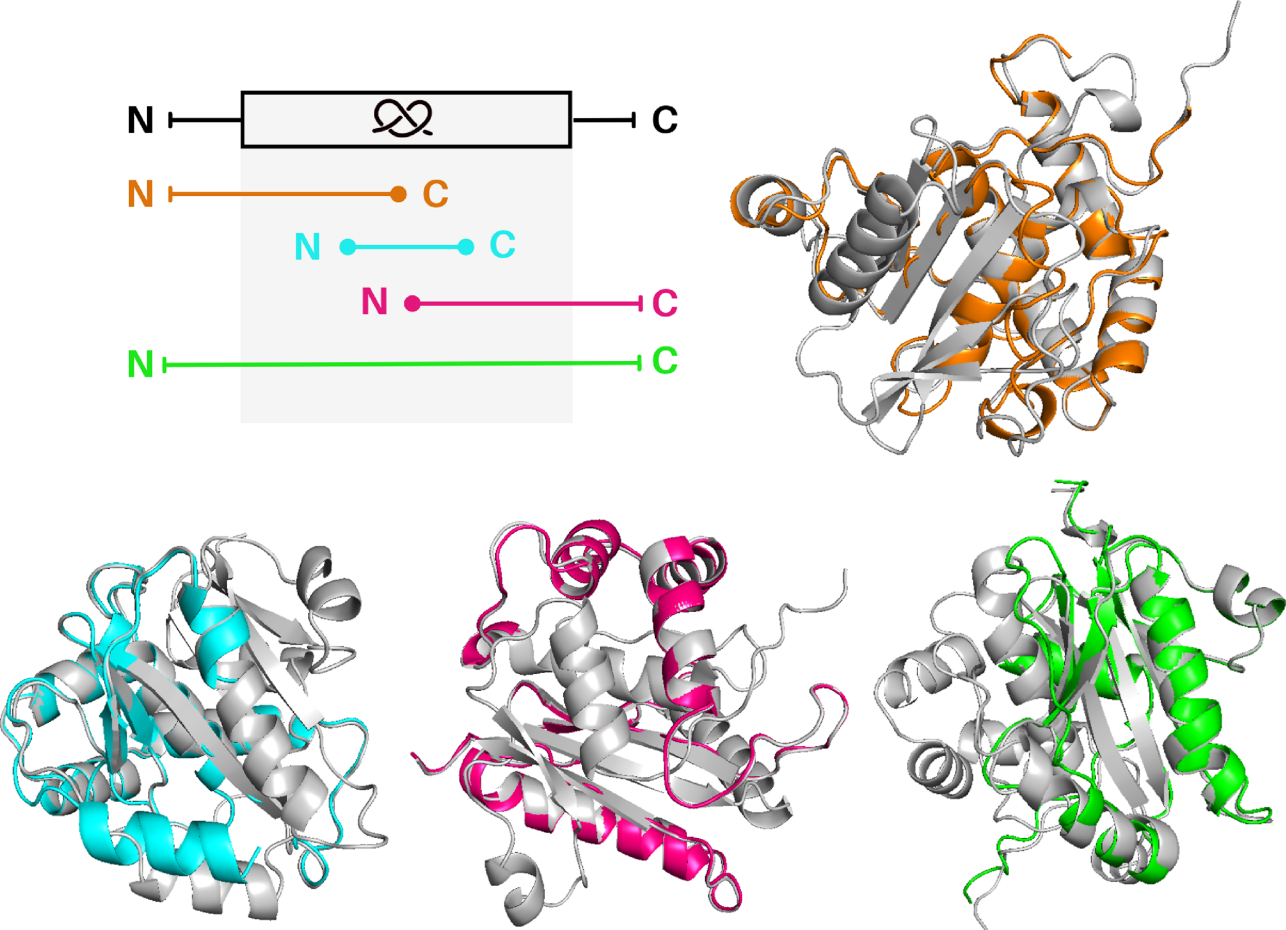
Unknotted UCH proteins superposed with their knotted homologs. Upper part: unknotted structure (orange; UniProtKB ID: A0A7S3I8E7) lacking the C‐terminal part of the knot core. Lower part: unknotted structure (cyan; UniProtKB ID: Q06AV7) without the N‐terminal and C‐terminal part of the knot core, unknotted structure (magenta; UniProtKB ID: A0A1A7YJ99) without the N‐terminal part of the knot core, and unknotted structure (green; UniProtKB ID: A0A024WZA) with low‐quality part of the knot core. All knotted homologs are shown with gray color (UniProtKB IDs: A0A078B145, A0A6P6PV05, A0A1A7X9F0, and W7F0M6, respectively).

The analysis revealed that all 475 unknotted proteins from the SPOUT superfamily we investigated have a close homolog (with an average sequence identity of 80%) that is knotted. The majority of these proteins are shorter than their knotted counterparts and the part that is lacking is always connected to the knotted region (Figure 5a). Predominantly, they lack the C‐terminal part of the knot core, but in some instances, they also miss the N‐terminal part or even the entire knotted region. However, the superposition of these structures shows a high level of similarity (Figure 5a). Adding the high sequence identity and the fact that more than 75% of these proteins are annotated as fragments (based on the InterPro database), we conclude that these are not truly unknotted proteins, but only fragmentary sequences found in a database (Figure 6).

However, we found 21 unknotted SPOUT proteins (Figure 2) that can be globally aligned with their knotted homologs. In 11 cases there is a deletion inside the knot core (Figure 5b). For the SPOUT proteins, such deletion means loss of function (Perlinska, Kalek, et al., 2020; due to a change in the shape of the active site, and lack of substrate‐binding residues), thus we do not suggest them as candidates for truly unknotted proteins. The next 7 proteins do not possess a knot but a slipknot topology due to the position of their C‐terminus in the AF model (Figure 5b). The slipknot topology has a “hidden” knot that can only be detected when analyzing the subchains of a structure. Here, we determined the topology of full‐chain models and, thus were not able to detect slipknots, which are in this case indistinguishable from the unknots. Due to the intact structure of a knot in these proteins, we believe, that they are knotted and their different topologies are a matter of modeling accuracy. The remaining cases are unknotted due to low‐quality knot fragments in the model (2) or are deleted entries (1). Therefore, we did not encounter any unknotted and functional methyltransferases in the SPOUT superfamily (Table 4).

**TABLE 4:** Unknotted proteins in knotted families.

Protein superfamily	No. analyzed proteins	Annotated as fragments (%)	No. truly^a^ unknotted proteins	Example
SPOUT	475	75	0	‐
Sodium/calcium exchanger	649	56	8	A0A2G1VBT6
UCH	83	92	6	A0A6J0D8H7
DUF2254 membrane	359	11	323	A0A1C5G9W9
DUF4253	27	4	26	A0A1I2KHH8

*Note*: Some portions of the proteins we analyzed are labeled as fragmentary in the InterPro database.

^a^ We define here truly unknotted proteins as those having a sequential equivalent to the knotted region.

A similar trend was observed for the other superfamilies in our dataset: sodium/calcium exchanger, AdoMeT synthase, Calcium‐activated potassium channel BK (alpha subunit), carbonic anhydrase, ribosomal protein L37/S30, and OPT.

#### 2.7.2 Carbonic anhydrase

All, except two members of the carbonic anhydrase family labeled as unknotted, have close entangled homologs (with an average sequence identity of 80.27%). 43% of the analyzed proteins lack the N‐terminal part and 57% of them miss the C‐terminal part, which in both cases are crucial to create a knot. Additionally, 66% of the unknotted proteins are annotated as fragments.

#### 2.7.3 AdoMet synthase

In this case, all unknotted proteins have a close‐knotted homolog with an average sequence identity of 80.1%. Considering 96% of the analyzed proteins either lack a C‐terminal or N‐terminal part and 82% of them are annotated as fragments, the AdoMet synthase superfamily almost certainly does not contain truly unknotted proteins.

#### 2.7.4 Calcium‐activated potassium channel BK (alpha subunit)

All unknotted, except one, members of this family are homologous to a knotted protein with an average sequence identity of 65.7%. After aligning corresponding homologs, it turns out that the unknotted proteins either lack a C‐terminal or N‐terminal part of a knot core, with a majority of the latter (78%). Also, in this case, we conclude that unknotted structures are only fragments of full proteins.

#### 2.7.5 Oligopeptide transporter

Similarly to the previous family, all unknotted proteins except one contain a homolog with a nontrivial topology (with an average sequence identity of 67.3%). 59.5% of the alleged unknotted OPTs lack the C‐terminal part of the knot core, 25% miss the N‐terminal part, and the rest lack both ends. Therefore, we assume that all members of this family are supposed to contain a knot.

#### 2.7.6 Ribosomal protein L37/S30

Finally, in the case of the Ribosomal protein L37/S30 family, all of its unknotted members contain a knotted homolog (with an average sequence identity of 64.1%). The majority of the unknotted proteins miss the C‐terminal part (57%) and the rest lack the N‐terminal part, essential to create entanglement.

#### 2.7.7 TDD

All unknotted, except two, TDD proteins have an entangled homolog (with an average sequence identity of 67.8%). While 58% of the unknotted members of this family either miss the N‐terminal or C‐terminal part of the knot core, the rest of the analyzed proteins seem to align well with both ends of their knotted homologs. Upon closer analysis, similarly, as for some of the proteins in the SPOUT superfamily, the reason for the lack of entanglement of the TDD members is caused by the deletion inside of the knotted region. Due to the substantial similarity between TDD and SPOUT proteins (e.g., identical knot structure), such deletion will negatively impact protein function, therefore, we did not find any promising candidates for truly unknotted proteins in this family.

### 2.8 Other cases

#### 2.8.1 Lantibiotic dehydratase

Based on our analysis, for the knot to occur in the LANTIBIOTIC dehydratases they additionally have to possess a thiopeptide‐type bacteriocin biosynthesis domain (IPR023809). In this case, none of the members labeled as unknotted possessed the aforementioned domain.

### 2.9 Substrate binding site important for knot detection

Finally, we return to ML investigation and ask whether the unique set of amino acids responsible for knot formation in the native state of protein can be detected. In order to determine what specific regions of proteins play a role in knot formation, we assessed the significance of continuous parts of the protein sequences (referred to as “patches”) using the model to obtain information about their importance on the prediction. This process is explained in detail in Section 5. Next, we focused on the most significant patches which we found in the largest group of knotted proteins, the SPOUT superfamily. Given that the average knot size in these proteins is about 47 amino acids, we chose patch lengths of 10, 20, and 40 residues. Our results suggest that longer patches, on average, hold more significance (Table 5).

**TABLE 5:** Percentage of proteins with a patch located in the knot core.

Patch size	Significant (%)	Not significant (%)
10	55	24
20	84	7
40	95	2

*Note*: Only patches that have at least 50% of their length inside the knot core were calculated.

**FIGURE 7:**
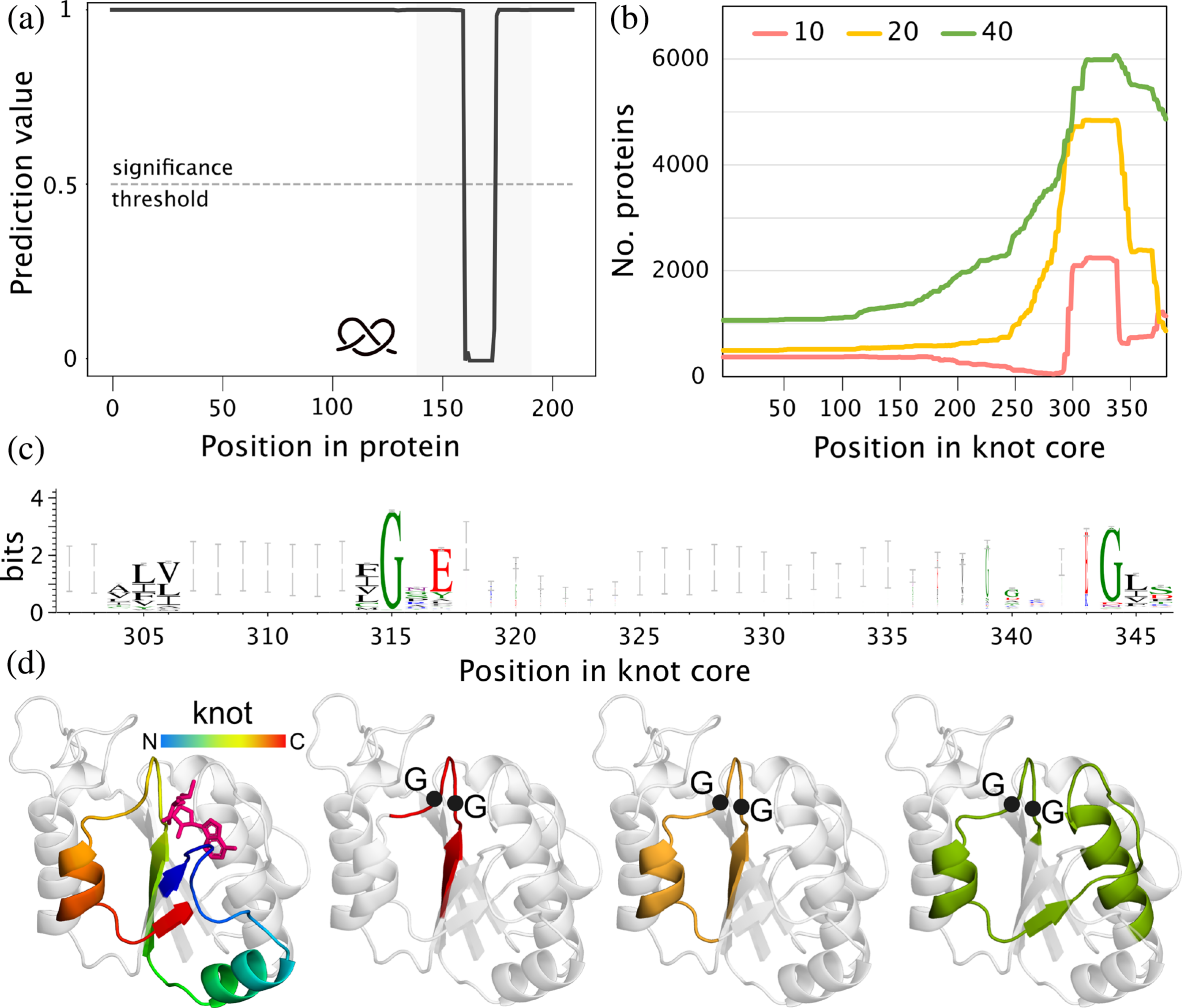
Important regions in SPOUT proteins based on machine learning model. (a) An example of how the model's prediction changes with respect to the patch location based on Nep1 protein (patches of length 10; UniProtKB ID: Q57977). Note the one significant drop inside the knot core. (b) Cumulative positions of the patches (with different lengths: 10, 20, and 40 amino acids) based on the knot core alignment. (c) Logo of the C‐terminal part of the knot core showing the conserved glycine motif. (d) Positions of patches in a TrmD structure (based on the N‐terminal domain of AlphaFold model of UniProtKB ID: B6YQ31). Colored regions represent from the left: knotted region (rainbow) and estimated location of the ligand (magenta); 10 (red), 20 (yellow), and 40 (green) amino acid‐long patches, respectively. Black dots show the position of the conserved glycine residues.

We started the analysis by localizing the positions of these patches in the protein sequences. This revealed a significant over‐representation in a specific region—the knot core. For instance, more than 80% of the patches of length 20 are found within the knotted region. Considering that most of these regions have <50 amino acids, and the proteins are on average 250 amino acids long, this accumulation within a specific region suggests that this region could be important for knot formation. Additionally, regardless of their length, all patches point to the same location within the knot—its C‐terminal part (Figure 7b). Based on the multiple sequence alignment of the knot cores, we observed that this region is the most conserved part of the knot, containing the glycine motif responsible for substrate binding (Figure 7c). This motif is also present in patches of lower significance, especially those of length 10. However, the conserved glycines are not a unique feature of knotted methyltransferases. They are also found in proteins with other folds, such as the Rossmann fold, which are unknotted (Perlinska, Stasiulewicz, et al., 2020). To conclude, in order to find an unambiguous set of amino acids unique to making a knot, one would have to analyze the family with knotted and unknotted homologs. However, the ATC/OTC family and the two new ones DUF2254 and DUF4253 have too few knotted sequences to constitute a sufficient dataset after clustering. Thus, this topic remains open.

## 3 DISCUSSION

In this study, we have assembled the largest dataset of knotted and unknotted proteins by analyzing AF's protein structure predictions using Topoly. Built upon this dataset, we have presented a novel transformer‐based ML model able to predict the knotting status only from the amino acid protein sequence. The model demonstrated robust performance across selected protein families, indicating its effectiveness in predicting the presence of knots on a large scale. Moreover, an additional evaluation made on a set of proteins whose structures were not predicted by AF also gives good results (92% correct predictions). We verified that this high performance was not correlated with high identity between tested proteins and the train set since there are numerous examples of correct predictions and low identity between the sets (<30%). The high level of convergence of our model's results with AF's predictions allows for its potential use as a quick way to obtain information about the topology of a given protein based on sequence alone. However, the training scheme with balanced dataset classes limits the model's performance on tasks such as scanning the whole UniProt database, where it can behave more like a sensitive sifter for potential knots requiring additional analysis. Also, the performance of the model outside its domain, that is, on other protein families, is not guaranteed. Results for one such family, ketol‐acid reductoisomerases with $4_{1}$ knot, can be found in Table S1. The model showed worse performance with a high false positive rate, however, it can be additionally fine‐tuned to be able to recognize a newly selected family. The limited power of the built model however does not hurt the idea of creating it as an intermediary for the data interpretation part.

To facilitate easy access to our model, we provide the link to the Hugging Face hub (https://huggingface.co/roa7n/knots_protbertBFD_alphafold) containing the trained model, along with scripts for creating the dataset, model training, and interpretation (https://github.com/ML-Bioinfo-CEITEC/pknots_experiments).

Subsequent utilization of the proposed interpretation patching technique on the trained model allowed for a deeper analysis of individual proteins and potentially caching amino acid patterns associated with the presence of knots in those proteins. The patching analysis conducted on the SPOUT family found a pattern of amino acids potentially necessary for knotting but on the other hand, these amino acids are also a key area for the co‐factor binding site, essential for the family representatives' enzymatic function. To conclude, finding an unambiguous set of amino acids that make a knot, would require a family with mixed, knotted and unknotted, topology. At present such families (ATC/OTC family and new ones: DUF2254, DUF4253, UCH, and sodium/calcium exchanger) have too few knotted sequences to constitute a sufficient dataset after clustering. Thus, this topic remains open.

Determining all proteins potentially knotted in UniProt allowed us to analyze knotting from the structural biology point of view, and the conservation of knots in families. Many of the AF predicted structures possess a knot. In some cases, these proteins are topological outliers within their families. Given that the knot is a conserved feature of a protein family such outlier structure most likely is an artifact. We constructed our dataset to avoid such improbable knotted structures and focused on those that form a bigger group. We found that knotted proteins predicted by AF can be clustered only into 15 superfamilies. They are listed in Table 1 plus two other families such as ketol‐acid reductoisomerases with $4_{1}$ knot and Halocarboxylic acid dehydrogenase DehI with $6_{1}$ knot.

Each family surprisingly includes both knotted and unknotted members. However, we found that there are only three new families indeed with knotted and unknotted members. In the case of the newly discovered DUF4253 family, sequence deletion within the knot core explained the trivial topology. For DUF2254, we observe the creation of a loop—which tied results in an unknot. In the case of the unknotted UCH proteins, they are analogous in structure to their knotted counterparts and the different topologies are due to a deletion in the N‐terminus that results in a trivial structure. Sequential clustering and structural verification revealed that both topologies are highly conserved within those families. All other families, however, appear to have only knotted proteins. Thus, unknotted proteins from known knotted families are typically nonfunctional fragments. Even though a significant portion of proteins is not annotated as fragments we show this explicitly in the case of the SPOUT superfamily, transmembrane, AdoMeT synthase, Calcium‐activated potassium channel BK (alpha subunit), Carbonic anhydrase, Ribosomal protein L37/S30, and OPT superfamilies that unknotted proteins in these families lack at least a portion of the knot core and are thus nonfunctional. Another reason for which the protein may be predicted as unknotted is the amino acid deletion in the knotted region as we have shown in the case of the TDD family.

To conclude, the study we performed has three main results. First, we created a dataset of knotted and unknotted proteins of sufficient size for Big Data analysis with the ML approach. This dataset may be utilized further in other studies requiring topologically distinct protein sequences. Secondly, we verified that the AF correctly predicts structures of proteins in our 15 biggest knotted families, including the less probable unknotted ones. We show, that the unknotted models are correct from the structural point of view. Thirdly, the knot is strictly conserved in 12 of the 15 knotted families. Unknotted models in these families are fragments of full‐length knotted proteins and not functional proteins with an alternative fold and topology.

## 4 CONCLUSIONS

Our systematic assessment and analysis of the AF database's protein structure predictions with regard to nontrivial topologies revealed that at least 0.3% of proteins are potentially entangled.

We used this data to show that it is possible to construct a large‐scale deep learning model that successfully classifies proteins from selected protein families as either knotted or unknotted based on their amino acid sequences. In the UniProt Database, there are around 50 million proteins without a structure predicted by the AF model (around 20%). Based on 100 selected proteins, whose structures were not predicted by AF, we have shown that our ML model also gives good results (92% correct predictions). Thus, the ML model could be used as a quick way to obtain information about the topology of a given protein based on sequence alone (e.g., for protein not predicted by AF) and without counting polynomials.

Furthermore, our application of interpretive techniques has illuminated specific patterns associated with conserved knots in SPOUT family proteins. These patterns are particularly pronounced in the areas of the cofactor binding site, a region crucial to the enzymatic function of these proteins. Our findings underscore the importance of this knot in maintaining protein function and raise interesting questions about the role of knotted structures in other protein families.

From the point of view of structural biology, we found that all potentially knotted proteins from UniProt can be classified only into 15 superfamilies. In these families, the same type of nontrivial topology is found in a significant number of homological sequences. This allows us to discover the presence of unknotted proteins in families with a highly conserved knot. Until now, such a situation has been observed for only one family (ATC/OTC). Herein, we have shown the potential existence of new families composed of knotted and unknotted proteins: UCH superfamily, sodium/calcium exchanger family, DUF2254 (transmembrane fold) and DUF4253 (globular fold).

Moreover, we prove that in the case of 13 out of 15 families, the knot is strictly conserved in the biologically functional proteins in a given family. The unknotted members predicted by AF are indeed unknotted but are created based on a fragmented protein sequence that lacks parts of the knot.

In summary, this has led to significantly advancing our understanding of the diversity and conservation of knotted protein families. On the other hand, we showed that AF correctly predicts structures of proteins in our dataset including the less probable unknotted ones. Finally, we created a dataset of knotted and unknotted proteins of sufficient size for Big Data analysis with the ML approach.

## 5 MODELS AND METHODS

### 5.1 Dataset

#### 5.1.1 Knot detection in AF database

With the introduction of AF database version 3, which for the first time revealed predictions of all protein structures from the UniProt database (nearly 200 million entries), knot detection and identification entered the Big Data age. Despite the relative ease of detecting a knot in a single structure using the Topoly Python package (Dabrowski‐Tumanski et al., 2020), the computational demands scale with the data size. Therefore, to address the needs and manage the size of computations an asynchronous distributed task management solution—the kafka‐slurm‐agent (https://github.com/prubach/kafka-slurm-agent) was implemented earlier and for the purpose of this study significantly enhanced.

The knot detection process was divided into two stages. In the initial stage, protein structures were grouped into batches of 4000. Structures with an average pLDDT score of the full chain below 70 were discarded, and for the remaining, the HOMFLY‐PT polynomial was computed using 100 random closures with a maximum of 60 crossings, as described in (Freyd et al., 1985; Przytycki & Traczyk, 2016). This stage took ~2 weeks, with the kafka‐slurm‐agent's cluster agents distributing the batches over three Linux clusters, collectively consisting of more than 70 nodes and ~2000 cores. Approximately 700,000 structures with an unknot probability of <0.5 were selected for the next stage.

Stage 2 involved running the HOMFLY‐PT polynomial with 500 random closures. Additional metadata from UniProt, such as taxonomy and InterPro annotations, were obtained. The position of the knot core was determined using a fast heuristic algorithm, adapted from the one developed for the AlphaKnot database (Niemyska et al., 2022). In light of the clusters' unavailability, most of this stage was computed on 10 individual workstations managed by worker–agents from the kafka‐slurm‐agent package. Structures with an unknot probability remaining lower than 0.5 and the probability of a particular knot exceeding 0.4 were classified as knotted.

This two‐stage knot detection procedure was reapplied to the 9% of structures updated in AF version 4.

We acknowledge that the applied filter of the average pLDDT score of the full chain above 70 in general does not equal our dataset being free of incorrect structures and by extension incorrect topological assignments. Despite the high average score, structures might contain unstructured loops that sometimes could introduce new artificial crossings. At the time of preparing the dataset, we were not aware of a robust algorithm that allows for differentiating those two types of loops (changing the topology or not). However, in further sections, we will focus on well‐defined and represented protein families, which will limit the amount of noise introduced to our model.

#### 5.1.2 Selection of protein families

Based on the resulting dataset of proteins with a detected knot in the predicted AF structure, proteins were categorized with respect to their assigned InterPro (Paysan‐Lafosse et al., 2022) identifiers (describing structural domains, families, and homologous superfamilies). Minimum subsets of such collections of identifiers were then selected, that is, the sets of the aforementioned structural feature identifiers whose representatives further manifested a nontrivial topology, in order to filter out redundant IDs. The dataset obtained was then subjected to quantitative search followed by manual verification, in which the most numerous groups (above 500 proteins) containing examples of proteins with experimentally resolved structures possessing a knot (based on the KnotProt database (Dabrowski‐Tumanski et al., 2018)) and groups of proteins with either close homology to experimentally confirmed knotted ones or new groups plausible to be knotted (as of the significant amount of nontrivial topologies) were chosen. Proteins that do not have the InterPro identifiers assigned or protein families with a low number of representatives were discarded. This ultimately produced a list of 20 different combinations of InterPro identifiers, included in Table 1. Due to the presence of a single domain in several different families, we found duplicate proteins in our dataset (Table S2). However, in further steps, we perform sequence clustering, eliminating the repeated protein sequences.

For each set of InterPro identifiers, we compiled a list of UniProt identifiers for the associated proteins. We then created a knotted dataset of protein sequences from the original AF calculation. We also created an unknotted dataset in a similar manner using the same collection of UniProtKB IDs, but we excluded the structures identified as potentially knotted in the first stage of knot detection to ensure accurate data labelling. Most of the data manipulation and processing were performed in‐house utilizing an installation of Apache Spark and Hadoop.

#### 5.1.3 Data preprocessing

To mitigate redundancy and prevent data leakage between the training and testing datasets, we adopted CD‐HIT (Fu et al., 2012), for clustering our protein sequence data prior to the training of the ML model. The CD‐HIT was configured with a sequence identity threshold of 90%, ensuring sequences sharing a local identity at or above this threshold were categorized into the same cluster. The minimum alignment coverage for the shorter sequences was set at 90%, and the maximum sequence length discrepancy was fixed at 80% of the length of the longer sequences. This ensured the inclusion of only those sequences with substantial alignment coverage. Following this, we chose a single representative sequence from each cluster with the rest of the similar proteins filtered out.

Given the discrepancies in length distributions between knotted and unknotted protein sequences, there was a risk that neural network models could unintentionally rely on sequence length as a classification feature. To address this concern, the datasets were subjected to downsampling, ensuring a balanced sequence length distribution between both protein sequence datasets.

The final dataset comprised 98,528 knotted proteins and an equivalent number of unknotted proteins. Sequences from each family were proportionally and randomly distributed into training and testing datasets with 157,644 (80%) samples designated for training and 39,412 samples (20%) set aside for testing.

#### 5.1.4 Selection of protein sequences without AF prediction

In order to assess the similarity between the training and verification set of proteins without AF prediction we used the “search” method from the MMseqs2 package (Steinegger & Söding, 2017). To ensure the global‐like alignment, coverage of at least 60% of the verification sequence was required, and the record with the highest identity level was selected. In total, 87 out of 100 proteins returned results, while for the other 13, no sequence from the training set passed the coverage threshold. Twenty‐six matched proteins had an identity level below 50% to their closest homolog.

#### 5.1.5 Analysis of families with potentially two topologies

For every unknotted protein we analyzed, its closest knotted homolog was found using a global–local algorithm GLSEARCH (e‐value $<1e^{-3}$). Based on their pairwise alignment the position of the unknotted protein was determined relative to the position of the knot core of the knotted protein. The knot cores were taken from the AlphaKnot database (Niemyska et al., 2022). Conservation of topology in the UCH, sodium/calcium exchanger, DUF4253 and DUF2254 domains was analyzed by performing gradual clustering using the “linclust” method from the MMseqs2 package (Steinegger & Söding, 2018). Sequences from the training set with aforementioned domains were clustered at the standard 80% both‐side coverage with the identity values ranging from 5% to 100%. Results suggest that 60% identity level is the optimal value with the highest frequency of uniform (trivial or knotted) clusters. Further structural analysis was performed with the “super” method from the PyMOL suite (Schrödinger, LLC, 2015).

### 5.2 Neural network models

Our primary objective was to engineer ML models capable of classifying knotted proteins based solely on their amino acid sequence.

The best‐performing model was based on the ProtBert‐BFD model architecture (Elnaggar et al., 2022), a derivative of the BERT language model that has been pre‐trained on a vast corpus of amino acid sequences, allowing it to capture important biophysical properties of the proteins.

The model and the tokenizer were downloaded from the Hugging Face Hub Rostlab/prot_bert_bfd repository. To determine the optimal parameters for training, we performed a hyperparameter search in relation to gradient accumulation, weight decay, and learning rate. The training set initially described was additionally further divided, with 90% of the sequences earmarked for actual training and the remaining 10% used as an evaluation set for comparing models trained with different sets of hyperparameters. The tuning with the best parameters resulted in a gradient accumulation = 16, weight decay = 0, and learning rate = 1*e*−5. Given the size of the dataset and acceptable convergence, we run only one training epoch. The model training required ~2.5 h on a single A100 GPU.

In addition to fine‐tuning 420 million trainable parameters of a large BERT‐like neural network model (M1), we also explored two alternate strategies: Employing protein‐level embeddings from the same model (420 M fixed parameters) and training a smaller neural network (16 M trainable parameters) on top of them (M2), and training a small convolutional neural network (M3) on a one‐hot encoded sequence (10 k trainable parameters). Refer to the Supplementary material S1 for a detailed description of models M2 and M3.

The models were implemented in Python using either Pytorch (Paszke et al., 2019; for M1, M2) or TensorFlow's Keras (Chollet, 2015; for M3). We leveraged the MetaCentrum computation cluster, which featured hosted JupyterLab notebooks and A10/A40/A100 GPUs, Python 3.8, PyTorch 1.13, TensorFlow 2.11, and HuggingFace transformers 4.24. For the code and the links to the trained models on Hugging Face Hub, please visit our GitHub repository: https://github.com/ML-Bioinfo-CEITEC/pknots_experiments.

All models were assessed employing standard accuracy metrics. To examine how the models performed across different protein families, and to ensure the models weren't solely recognizing a specific subset of families, we also calculated and monitored accuracy per family, true positive rate, and true negative rate with the classification threshold set to 0.5.

### 5.3 Interpretation with patching technique

The core problem we addressed was the binary classification of proteins as either knotted or unknotted. The secondary aim was to identify the crucial segments of protein sequences that largely contribute to the formation of a knot. Given the availability of the actual knot core locations within knotted sequences, we conducted an evaluation to measure the model's capability to not only correctly classify an input sequence, but also to pinpoint the knot core's location. However, conventional interpretability techniques, like Layer Integrated Gradients (Cik et al., 2021), have limited applicability on biological data since they can only relate to individual input points (in our case amino acids), whereas protein folding requires the cooperation of groups of them simultaneously. Therefore, we proposed a patching technique: we monitored how the model's prediction changed after replacing a continuous segment of amino acids in the original sequence with *X* characters, with respect to the model's prediction of the original unpatched sequence. The *X* character was chosen because it is present in the ProtBert‐BFD tokenizer and it signifies an arbitrary amino acid, and the patch size was set specifically for each protein family based on their average knot core and sequence lengths.

Our hypothesis is that if we patch a part of the sequence corresponding to the knot core, the prediction score will drop (from knotted to unknotted), reflecting the significance of such a segment. In practical terms, if the prediction score fell below 0.5, the corresponding patch was deemed a candidate for the knot core location. For each sequence, we generated its patched versions by moving the patch from left to right with stride 1 and then fed them to the model to obtain their predictions. The patch that resulted in the overall minimum prediction score among all patched versions was chosen as the predicted knot core location. This approach was evaluated by calculating the overlap of the minimum patch location with the actual knot core. Figure S2 illustrates this technique for one input sequence.

## AUTHOR CONTRIBUTIONS

**Maciej Sikora:** Data curation; investigation; writing—original draft; validation; writing—review and editing. **Eva Klimentova:** Investigation; writing—original draft; writing—review and editing; validation; visualization; software. **Dawid Uchal:** Investigation; writing—original draft; data curation. **Denisa Sramkova:** Investigation; writing—original draft; writing—review and editing; validation; visualization; software. **Agata P. Perlinska:** Investigation; validation; visualization; writing—review and editing; writing—original draft. **Mai Lan Nguyen:** Investigation; writing—original draft; validation; visualization; writing—review and editing. **Marta Korpacz:** Investigation; writing—original draft; writing—review and editing; validation; visualization. **Roksana Malinowska:** Writing—original draft; investigation; validation; visualization. **Szymon Nowakowski:** Validation. **Pawel Rubach:** Investigation; writing—original draft; data curation. **Petr Simecek:** Conceptualization; funding acquisition; writing—original draft; writing—review and editing; project administration; supervision. **Joanna I. Sulkowska:** Conceptualization; funding acquisition; writing—original draft; writing—review and editing; project administration; supervision.

## Supporting information


**Data S1:** Supporting Information.

**Data S2:** Supporting Information.
