# Supplementary material for: Knot or not? Identifying unknotted proteins in knotted families with sequence‐based Machine Learning model
Source: Protein Sci. 2024 Jun 18;33(7):e4998. doi: 10.1002/pro.4998 (PMC11184937; doi:10.1002/pro.4998)
Supplement: Supplementary file 2 — Data S2: Supporting Information. [file PRO-33-e4998-s002.pdf]

Knot or Not? Identifying unknotted proteins in knotted families with sequence-based ML model

Supplementary Material

Maciej Sikora*, Eva Klimentová*, Dawid Uchal, Denisa Šrámková, Agata P. Perlinska, Mai Lan Nguyen, Marta Korpacz, Roksana Malinowska, Szymon Nowakowski, Pawel Rubach, Petr Šimeček, Joanna I. Sulkowska

M2 model

The M2 model is a simple convolutional neural network (CNN) that leverages protein embeddings from the ProtBert-BFD model [1]. These embeddings are processed using average pooling to yield a fixed-size vector of 1024 dimensions. The CNN is composed of a convolutional layer with a kernel size of 7 and 32 channels, followed by batch normalization and a ReLU activation function. The output is then flattened and passed through two linear layers before being processed by a single neuron with a sigmoid activation function, yielding a probability of the input sequence being knotted.

The model was trained over 10 epochs using Adam optimizer with default settings, learning rate 0.0001 and a batch of size 32. The model reached 96.9 % accuracy on the test set.

M3 model

The M3 model, a CNN, is less complex compared to the other two, borrows its architecture from the PENGUINN model [2]. We selected training and testing data such that sequences exceeding 500 amino acids were filtered out. Sequences shorter than 500 amino acids were padded with the 'X' character to reach the uniform length of 500. The sequences were then one-hot encoded to serve as inputs to the model.

Training of the M3 model spanned over 10 epochs using the Adam optimizer with default parameters, a batch size of 128, and a validation split of 0.3. Upon evaluation of the test set, it achieved an accuracy of 95.18%.

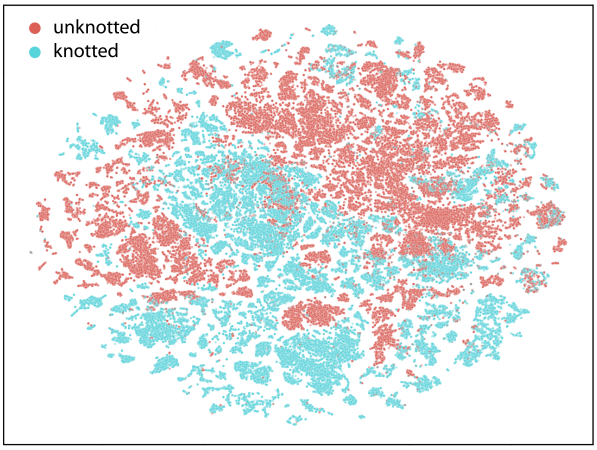

Figure 1: Visualization of protein sequences embeddings from ProtBert-BFD using t-SNE technique, coloured according to topology type in the training set.

DUF2254 and DUF4253 analysis

The topological conservation of proteins with either DUF2254 or DUF4253 domains was analysed with sequential clustering and verified structurally. The results have been summarised in the DUF_analysis.xlsx Spreadsheet. Data presents topological consistency of clusters at different values of identity. For the 60% threshold, we additionally present statistics of structural superposition. Finally, we report clusters containing both trivial and knotted proteins and outliers with high RMSD to other members. Additionally, the comparison between knotted and unknotted proteins is visualized in the main text in Figure 3.

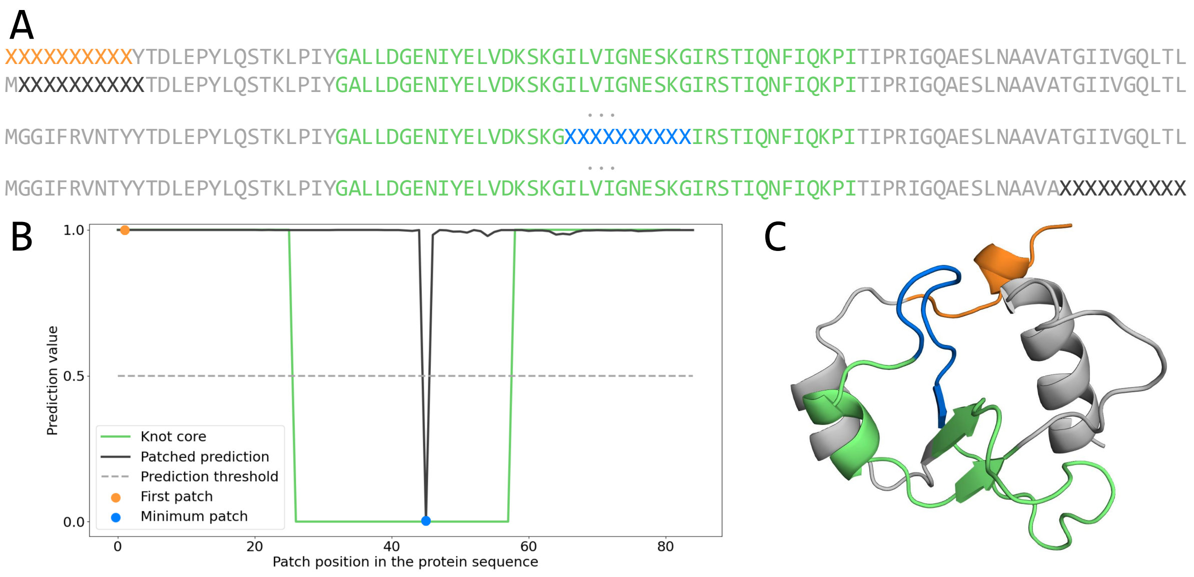

Figure 2: **The patching technique demonstrated on one SPOUT protein (UniProtKB ID: A0A2W5F4Z7).** A. The patched versions of the input sequence with two specific patches highlighted (first: orange, minimum: blue). B. A plot of the prediction scores for all patched versions of the sequence. We can observe one significant drop below the 0.5 (knotted to unknotted) threshold and its corresponding location (blue). C. The 3D protein structure with highlighted knot core (green), minimum patch (blue), and the first patch (orange).

Table 1: **Evaluation of models on ketol-acid reductoisomerases family.** False positive rate (FPR) and false negative rate (FNR) of six models evaluated on the test set of the ketol-acid reductoisomerase family.

Model	FPR (%)	FNR (%)
M1	57.94	8.44
M2	67.61	2.22
M3	34.64	77.22
M1b	8.24	1.33
M2b	2.84	8.00
M3b	3.88	6.74

Validation on ketol-acid reductoisomerases

For independent validation, we selected a family of ketol-acid reductoisomerases, excluded from the training set. Experimental verifications suggest that some proteins in this family possess a 4_1 knot [3]. The sequences were processed similarly to the main dataset (clustering, train/test split set at an 80:20 ratio). In total, we have 2304 proteins in the training set (794 knotted, 1510 unknotted) and 577 proteins in the test set (225 knotted, 352 unknotted).

Initially, we tested the pre-trained models M1, M2, and M3. As depicted, the accuracy of the predictions generally suffers for either positive or negative cases. This discrepancy likely stems from family-specific knotting features that the models had not been trained to detect. We can conclude, that the model cannot be relied on to predict the knots outside its domain (i.e. families it has been trained on). This discrepancy can be visualized as a T-SNE plot of protein embeddings, where predictions from M2 are on the left, and the true labels (knotted vs unknotted) are on the right, as shown in Supplementary Figure 3.

Not only do we observe that the current models are not performing well, but it is also evident the knotted and unknotted cases in Supplementary Figure 3 can be separated. For that, we fine-tuned M1, M2, and M3 specifically for this family, resulting in models M1b, M2b, and M3b. A comparison of false positive rates across all six models is displayed in Supplementary Table 1.

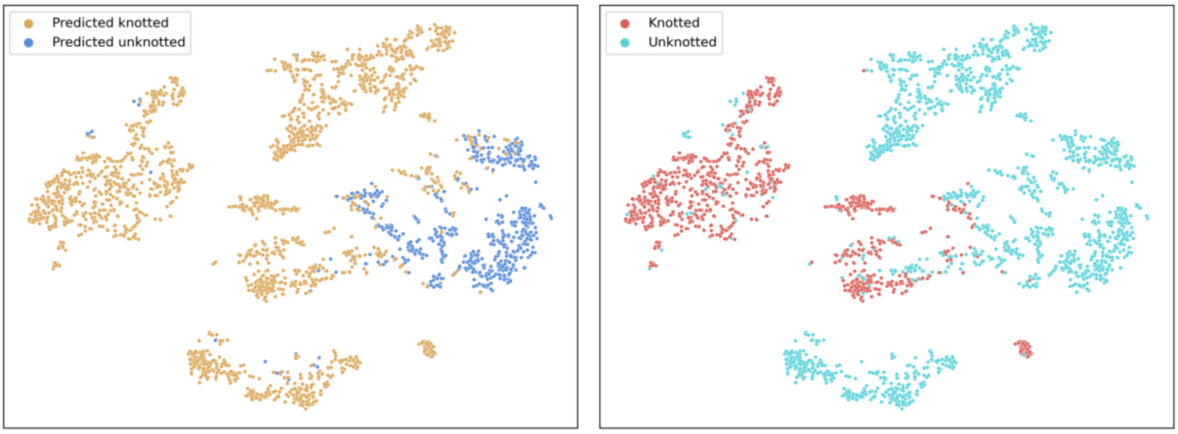

Figure 3: Visualization of protein sequence ProtBERT-BFD embeddings from the ketol-acid reductoisomerase family using t-SNE technique. The left panel is coloured according to M2 model predictions, the right panel is coloured based on true topology.

Table 2: **Overlap between IPR002035 (von Willebrand factor, type A) and other families/domains in our dataset.** Protein numbers are based on the UniProtKB.

Family ID	Family/domain name	No. proteins with both domains
IPR013694	VIT	18562
IPR032695	Integrin domain superfamily	6774
IPR013649	Integrin alpha-2	6318
IPR036398	Alpha carbonic anhydrase domain superfamily	15
IPR004837	Sodium/calcium exchanger membrane region	2
IPR029026	tRNA (guanine-N1-)-methyltransferase, N-terminal	1

Table 3: **Dataset of selected proteins without structure prediction in AlphaFold database.** For each protein, we search for its closest homolog and report its topology and sequence identity (% in parenthesis). The asterisk indicates that the query protein does not contain the knotted core of its homolog and, thus is likely an unknotted fragment. For the AlphaFold models we generated for each query protein, we report their topology and global pLDDT values (a value above 70 indicates the overall good quality of the model).

**Protein ID**	**Our model (score)**	**AlphaFold model**	**Max. identity to train set (%)**	**Homolog**	**Prediction**
IPR029026 (SPOUT)					
A0A8H3XI24	0.9976	3_1 (82.97)	30	3_1 (94.4)	correct
A0A2E9CFS3	0.7200	3_1 (73.48)	32.5	3_1 (31.1)	correct
A0A8T6ML81	0.3252	0_1 (79.14)	33.3	3_1 (32.2)	**incorrect**
A0A8J5EEA0	0.0013	0_1 (41.67)	32.7	3_1 (27.7) *	correct
A0A968VFV8	0.0003	0_1 (81.12)	29.7	3_1 (57.8) *	correct
IPR038459 (Trm10)					
A0A914JA29	0.9960	3_1 (73.41)	44.9	3_1 (34.9)	correct
A0A922L0N8	0.9980	3_1 (79.67)	38.2	3_1 (35.9)	correct
A0A9D4SDB1	0.9976	3_1 (79.04)	38.5	3_1 (36.2)	correct
A0A915PGF3	0.9927	3_1 (74.81)	67.3	3_1 (66.9)	correct
A0A8L7TK29	0.9927	3_1 (74.71)	100	3_1 (100)	correct
IPR002035 (von Willebrand)					
A0A8C2EGX6	0.0002	0_1 (68.7)	41.5	0_1 (74.9)	correct
A0A8S1V0J7	0.0008	0_1 (73)	47	3_1 (46.6) *	correct
A0A9K3KCI8	0.0004	0_1 (60.84)	26.4	-	correct
A0A8S1XFL7	0.0005	0_1 (73.52)	46.1	3_1 (82.9) *	correct
A0A8S1YLQ6	0.0004	0_1 (79.85)	78.2	3_1 (78.2) *	correct
IPR013694 (VIT)					
A0A956IK71	0.0004	0_1 (86.21)	76.5	3_1 (76.3) *	correct
A0A8C7XVN3	0.0031	0_1 (56.29)	50.7	0_1 (79.8)	correct
A0A956ZFB7	0.9990	3_1 (79.77)	-	3_1 (27)	correct
A0A9D7H5N9	0.0826	0_1 (88.84)	38.6	3_1 (38.5)	**incorrect**
A0A956IUH5	0.0005	0_1 (84.66)	61.7	3_1 (57.2) *	correct
IPR036901;IPR006131 (ATC/OTC)					
A0A8T4EC19	0.9995	3_1 (85.09)	54.2	3_1 (45.5)	correct
A0A8S1BF55	0.0001	0_1 (86.9)	52.6	0_1 (49.7)	correct
A0A9J7EFQ0	0.0002	0_1 (88.82)	69	0_1 (87.1)	correct
A0A8T4WQH7	0.9990	3_1 (91.98)	77.6	3_1 (77.7)	correct
A0A921Z5A2	0.0002	0_1 (90.08)	72.3	0_1 (72.4)	correct
IPR004837 (Membrane transporter)					
A0A8S1SUD9	0.9995	3_1 (85.24)	95.9	3_1 (96)	correct
A0A8S1SWC8	0.9995	3_1 (84.21)	91.7	3_1 (91.8)	correct
A0A8J5WIR3	0.9995	3_1 (82.85)	89	3_1 (90)	correct
A0A8J8CM25	0.9950	3_1 (76.13)	71.8	3_1 (68.1)	correct
A0A8X7XUV6	0.9990	3_1 (84.16)	97.5	3_1 (98.1)	correct
IPR022628;IPR002133;IPR022636 (SAM synthase)					
A0A928Y5T2	0.9960	3_1 (91.72)	100	3_1 (100)	correct
A0A9E5E0B7	0.9990	3_1 (95.11)	40.6	3_1 (41.2)	correct
A0A947UDQ2	0.9927	3_1 (95.09)	42.2	3_1 (42.4)	correct
A0A9D5JR46	0.9966	3_1 (93.06)	57.7	3_1 (57.6)	correct
A0A8S6XMR0	0.4119	3_1 (92.82)	53.7	3_1 (87)	**incorrect**
IPR027790;IPR042544;IPR002795 (SAM synthase)					
A0A8J8PY70	0.9917	3_1 (92.16)	88.7	3_1 (88.8)	correct
A0A9E5DMX5	0.9927	3_1 (95.52)	98.7	3_1 (98.8)	correct
A0A8T7GQY4	0.9927	3_1 (96.69)	83.2	3_1 (83.2)	correct
A0A8D6PNX3	0.9927	3_1 (96.29)	91.3	3_1 (94.1)	correct
A0A8D6T0V3	0.9927	3_1 (96.15)	91.8	3_1 (94.6)	correct

IPR036398 (Carbonic anhydrase)					
A0A8J2AY33	0.9854	3_1 (71.2)	-	3_1 (22.6)	correct
A0A9E5F7I8	0.0086	0_1 (52.98)	28.3	3_1 (25.1) *	correct
A0A2E4Q8P5	0.9790	3_1 (85.6)	56.6	3_1 (56.7)	correct
A0A966UFS7	0.9900	0_1 (82.55)	57.5	3_1 (31.4)	correct
A0A8S2FJT4	0.0020	0_1 (84.77)	-	0_1 (100)	correct
IPR018883 (Carbonic anhydrase)					
A7UC73	0.9990	3_1 (80.3)	81.4	3_1 (48.1)	correct
A0A8J2WXS8	0.9990	3_1 (81.87)	65.9	3_1 (100)	correct
A0A9K3PHQ4	0.9990	3_1 (84.29)	63.9	3_1 (57.5)	correct
A0A9K3LKP9	0.9990	3_1 (84.42)	62.9	3_1 (58)	correct
A0A8J3EH14	0.9990	3_1 (81.73)	75.8	3_1 (68.8)	correct
IPR004813 (OPT transporter)					
A0A970JNY6	0.9995	5_2 (92.9)	87.2	3_1 (87.2)	correct
A0A8T5MWS8	0.0004	0_1 (80.99)	83	3_1 (83) *	correct
A0A954DGS6	0.0006	0_1 (91.31)	64.9	3_1 (64.3) *	correct
A0A8T3NJ97	0.0007	0_1 (88.6)	64.2	3_1 (68.2) *	correct
A0A8T5MY29	0.0004	0_1 (84.69)	91.3	3_1 (91.4) *	correct
IPR032695;IPR013649 (Integrin alpha)					
A0A8K0KI73	0.3884	3_1 (71.5)	26.8	3_1 (25.1)	**incorrect**
A0A8J7NT24	0.0080	0_1 (56.97)	-	0_1 (28.6)	correct
A0A9D3Y583	0.2646	0_1 (78.61)	34.9	0_1 (38.4)	correct
A0A8U0Q3E6	0.6790	0_1 (76.18)	94.4	0_1 (91.8)	**incorrect**
A0A974C0Z4	0.0026	0_1 (85.86)	40.5	0_1 (100)	correct
IPR005636 (Acp transferase)					
A0A8J2ENG2	0.9976	3_1 (64.1)	24	-	correct
A0A8J4LW13	0.9990	3_1 (66.3)	-	3_1 (54.1)	correct
A0A2C6KEV2	0.9976	3_1 (56.91)	-	3_1 (35.6)	correct
A0A915AWJ5	0.9990	3_1 (65.82)	93.1	3_1 (37.5)	correct
A0A8J4BSF7	0.9990	3_1 (59.57)	-	3_1 (49.5)	correct
IPR007209;IPR022968;IPR007177 (Acp transferase)					
A0A8H7ZWX2	0.9990	3_1 (48.9)	-	3_1 (30)	correct
A0A8I3PQD2	0.9990	3_1 (65.2)	94.6	3_1 (95.8)	correct
A0A8P0SNY3	0.9990	3_1 (65.11)	94.6	3_1 (95.8)	correct
A0A8H7IL29	0.9980	3_1 (65.8)	86.9	3_1 (89.7)	correct
A0A8K0LDV6	0.9990	3_1 (68.15)	56.8	3_1 (68.9)	correct
IPR003929 (Ion channel)					
A0A8B7E2W2	0.0629	0_1 (81.06)	71.5	3_1 (52.2) *	correct
A0A7R8H1T0	0.0009	0_1 (65.78)	58.3	3_1 (56.7) *	correct
A0A8C2XNN4	0.0053	0_1 (67.16)	88.3	0_1 (89.2)	correct
A0A8J4W530	0.0022	0_1 (78.58)	98.7	0_1 (100)	correct
A0A8C0GE48	0.0014	0_1 (68.62)	70.8	3_1 (79.5) *	correct
IPR001578;IPR036959 (UCH)					
A0A8E2EBJ8	0.9980	5_2 (78.67)	48.8	5_2 (41.5)	correct
A0A8C2NVE0	0.0027	0_1 (57.13)	-	5_2 (90.3) *	correct
A0A8H4Y5L3	0.9976	5_2 (80.45)	69.9	5_2 (72)	correct
A0A8H7NIG0	0.9976	5_2 (79.22)	99.7	5_2 (99.8)	correct
A0A8S4HBC1	0.9910	5_2 (81.25)	96.4	5_2 (99.2)	correct
IPR018723 (DUF2254)					
A0A932D1B6	0.0013	0_1 (92.4)	58	0_1 (57.5)	correct
A0A961TQP8	0.0013	0_1 (84.46)	49.2	0_1 (51.6)	correct
A0A8J3IFF8	0.0007	0_1 (82.94)	47.8	0_1 (51.2)	correct
A0A8E4P880	0.9980	3_1 (84.09)	77.1	3_1 (89.3)	correct
A0A924UVV1	0.0023	0_1 (86.42)	80	0_1 (97.6)	correct
IPR006827 (Lantibiotic dehydratase)					
A0A9K3TKK5	0.0004	0_1 (92.87)	100	0_1 (99.8)	correct
A0A925GID4	0.0004	0_1 (88.96)	49.7	0_1 (49.5)	correct

A0A962IAM5	0.0003	0_1 (86.79)	32.1	5_2 (34.8) *	correct
A0A925RNK6	0.0205	0_1 (92.58)	72.3	0_1 (72.1)	correct
A0A5C2D042	0.0003	0_1 (88.87)	45.3	0_1 (44.6)	correct
IPR010793 (Riboproteins)					
A0A8D2JY13	0.9307	0_1 (78.51)	97.5	3_1 (98.3)	correct
A0A9J7LYR8	0.9937	3_1 (73.07)	100	3_1 (84.8)	correct
A0A8C2NS88	0.0176	3_1 (84.42)	78.5	3_1 (74)	**incorrect**
A0A8J9VB01	0.9940	3_1 (75.31)	91.1	3_1 (87.1)	correct
A0A8J6GTM5	0.9750	3_1 (80.61)	93.2	3_1 (80.5)	correct
IPR025349 (DUF4253)					
A0A939XUL2	0.9473	3_1 (68.5)	-	3_1 (25.5)	correct
A0A956ZEP6	0.9927	0_1 (58.73)	-	0_1 (25.5)	**incorrect**
A0A9E1HYU6	0.9750	3_1 (95.46)	82.2	3_1 (93.2)	correct
A0A8J8AKZ2	0.9946	0_1 (53.07)	-	0_1 (22.1)	**incorrect**
A0A956QTU2	0.0097	0_1 (79.31)	-	0_1 (25.7)	correct

References

[1] A. Elnaggar, M. Heinzinger, C. Dallago, G. Rehawi, Y. Wang, L. Jones, T. Gibbs, T. Feher, C. Angerer, M. Steinegger, D. Bhowmik, and B. Rost, "Prottrans: Toward understanding the language of life through self-supervised learning," *IEEE Transactions on Pattern Analysis and Machine Intelligence*, vol. 44, no. 10, pp. 7112–7127, 2022.

[2] E. Klimentova, J. Polacek, P. Simecek, and P. Alexiou, "PENGUINN: Precise exploration of nuclear g-quadruplexes using interpretable neural networks," *Front. Genet.*, vol. 11, p. 568546, Oct. 2020.

[3] P. Virnau, L. A. Mirny, and M. Kardar, "Intricate knots in proteins: Function and evolution," *PLOS Computational Biology*, vol. 2, no. 9, p. e122, 2006.
